# Tailored Fluorosurfactants through Controlled/Living Radical Polymerization for Highly Stable Microfluidic Droplet Generation

**DOI:** 10.1002/anie.202315552

**Published:** 2023-12-12

**Authors:** Xiangke Li, Shi‐Yang Tang, Yang Zhang, Jiayuan Zhu, Helen Forgham, Chun‐Xia Zhao, Cheng Zhang, Thomas P. Davis, Ruirui Qiao

**Affiliations:** ^1^ Australian Institute of Bioengineering and Nanotechnology The University of Queensland Brisbane, Queensland 4072 Australia; ^2^ School of Electronics and Computer Science University of Southampton Southampton SO17 1BJ UK; ^3^ School of Engineering, Faculty of Science and Engineering Macquarie University Sydney, NSW 2109 Australia; ^4^ School of Chemical Engineering and Advanced Materials The University of Adelaide Adelaide, SA 5005 Australia

**Keywords:** Droplet Microfluidics, Fluorosurfactant Perfluoropolyether, Living Radical Polymerization

## Abstract

Droplet‐based microfluidics represents a disruptive technology in the field of chemistry and biology through the generation and manipulation of sub‐microlitre droplets. To avoid droplet coalescence, fluoropolymer‐based surfactants are commonly used to reduce the interfacial tension between two immiscible phases to stabilize droplet interfaces. However, the conventional preparation of fluorosurfactants involves multiple steps of conjugation reactions between fluorinated and hydrophilic segments to form multiple‐block copolymers. In addition, synthesis of customized surfactants with tailored properties is challenging due to the complex synthesis process. Here, we report a highly efficient synthetic method that utilizes living radical polymerization (LRP) to produce fluorosurfactants with tailored functionalities. Compared to the commercialized surfactant, our surfactants outperform in thermal cycling for polymerase chain reaction (PCR) testing, and exhibit exceptional biocompatibility for cell and yeast culturing in a double‐emulsion system. This breakthrough synthetic approach has the potential to revolutionize the field of droplet‐based microfluidics by enabling the development of novel designs that generate droplets with superior stability and functionality for a wide range of applications.

## Graphical abstract

Droplet‐based microfluidics reimagined using LRP‐synthesized fluorosurfactants. Improved stability, biocompatibility, and promising applications.

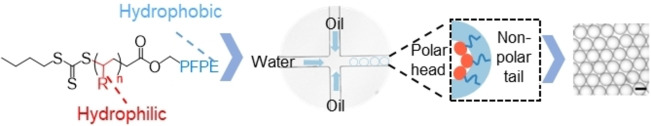

## Introduction

Droplet microfluidics is a powerful tool used in bioanalytics to generate and manipulate small‐volume drops ranging from picoliters to nanoliters in microscale channels.^[1]^ This approach has enabled innovative research and product development in the fields of chemistry and biology,^[2]^ including applications such as small‐scale organic synthesis,^[3]^ single‐cell sequencing,^[4]^ directed enzymatic evolution,^[5]^ high throughput drug screening,^[6]^ and digital polymerase chain reaction (PCR).^[7]^ In most cases, the use of surfactants is crucial to prevent coalescence between droplets and stabilize their formation.^[8]^ However, despite its advantages, formulating emulsified droplets with optimal performance for biological applications remains a significant challenge due to the lack of proper surfactants.^[9]^

Fluorosurfactants, also known as fluorinated surfactants, are often dissolved in biologically inert fluorinated oil solvents to create water‐in‐fluorinated‐oil (w/o) emulsions.^[8]^ These emulsions have the added benefit of the fluorinated oil's ability to hold dissolved oxygen, making them useful for cell culture and other biological applications.^[10]^ Fluorosurfactants typically consist of a fluorous ‘tail’ group and a hydrophilic ‘head’ group, which work together to reduce the surface tension at interfaces between water and oil.^[9a]^ Perfluoropolyether (PFPE) is often used as the primary component of the fluorous ′tail′ because of its excellent solubility in fluorinated oil and its capacity to provide effective and long‐term stabilization. To ensure biocompatibility, the surfactant's hydrophilic head group should not interfere with the biological or chemical processes occurring inside the droplet.^[9b]^ For this reason, biocompatible moieties, such as polyethylene glycol (PEG), have been utilized in droplet‐based microfluidics to mitigate potential interference.^[11]^ For example, the commercially available tri‐block copolymer fluorosurfactants, PEG‐PFPE_2_ (EA surfactant from RAN Biotechnologies, Billerica, Massachusetts, USA), and the structurally related derivate Jeffamine‐PFPE_2_ (Pico‐Surf^TM^, Dolomite Microfluidics, Royston, UK) have been widely used for droplet stabilization in fluorinated oil.[^10^, ^11^, ^12^] However, the currently available state‐of‐the‐art surfactants synthesized through the reaction between PFPE and PEG often involve complex synthetic procedures and yield low productivity. This complex synthetic route can result in the formation of unwanted impurities, including di‐block, unmodified precursors, and ionically coupled surfactant molecules.^[1]^ Moreover, the modification of these surfactants with other functional groups, such as catalysts, receptors/ligands, or fluorophores, is limited, thereby greatly restricting their potential for more advanced applications.^[13]^

Herein, for the first time, we report on a novel and highly efficient approach based on controlled living radical polymerization (LRP) for synthesizing customized fluorosurfactants that demonstrate exceptional performance in providing ultra‐high stability for droplets created in different aqueous solutions including water, biological media such as phosphate‐buffered saline (PBS) and cell culture medium. Compared to the commercialized surfactant Pico‐Surf^TM^, our surfactants outperform in thermal cycling for PCR testing, and exhibit exceptional biocompatibility for cell and yeast culturing in a double‐emulsion system. This breakthrough synthetic approach has the potential to revolutionize the field of droplet‐based microfluidics, enabling the development of novel designs that generate droplets with superior stability and functionality for a wide range of applications.

## Results and Discussion

### Synthesis of fluorosurfactants using LRP

Controlled/living radical polymerization (LRP) offers several advantages over conventional free radical polymerization techniques.^[14]^ First and foremost, LRP allows for precise control over the molecular weight and composition of the polymer product, resulting in polymers with narrow polydispersity and well‐defined end groups. Another advantage of LRP is that it can be used to polymerize a wide range of functional monomers, including those that are typically difficult to polymerize using conventional radical polymerization methods.^[15]^ This versatility enables the synthesis of polymers with diverse chemical and physical properties in a controlled manner.^[16]^

**Figure 1:**
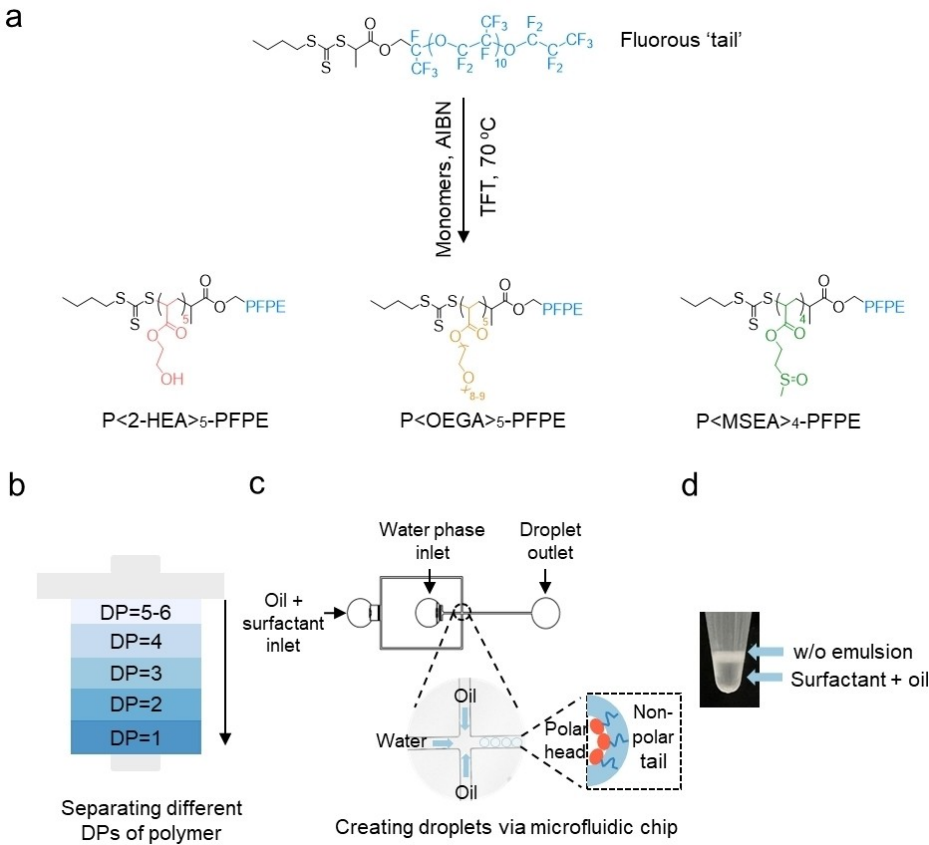
Synthesis of fluorosurfactants using RAFT polymerization. a) Synthesis of PFPE‐containing parent polymers of 2‐HEA, MSEA and OEGA fluorosurfactants. b) Schematic diagram of flash chromatographic separation of parent PFPE polymers with an uneven distribution of chains into several distinct fractions with various compositions and PFPE content was decreased within 7 column volumes. c) Schematic diagram of the polydimethylsiloxane (PDMS) microfluidic devices for droplet generation. Channel width: 100 μm, height: 50 μm. d) Representative image of generated droplets using P<2‐HEA>_5_‐PFPE surfactant within 1.5 ml tube.

In the current study, we synthesized a series of PFPE‐based fluorosurfactants using reversible addition fragmentation chain transfer (RAFT) polymerization, one of the most versatile LRP methods. Three distinct hydrophilic monomers were used, i.e., oligo ethylene glycol acrylate (OEGA), 2‐hydroxyethyl acrylate (2‐HEA), and 2‐(methylsulfinyl)ethyl acrylate (MSEA) (Figure 1a), to evaluate their performance as surfactant for stabilizing water‐in‐oil (w/o) droplets. 2‐(butylthiocarbonothioylthio)‐propionic acid (BTPA)–PFPE macro‐chain transfer agent (macro‐CTA) was firstly synthesized through an esterification coupling reaction using N‐(3‐(dimethylamino)propyl)‐N′‐ethylcarbodiimide hydrochloride/4‐(dimethylamino)pyridine (EDCl/DMAP) as described in our previous work.^[17]^ Both ^1^H and ^19^F NMR spectra indicate the successful preparation of macro‐CTA (Figure S1). Both the 2‐HEA and OEGA monomers are commercially available, while the MSEA monomer was synthesized according to our previous work^[18]^ and characterized by the ^1^H NMR spectra (Figure S2). The monomers were then polymerized through a single step RAFT polymerization reaction using azoisobutyronitrile (AIBN) as the initiator. The resulting parent polymers, (annotated as P<2‐HEA>_5_‐PFPE, P<OEGA>_5_‐PFPE, and P<MSEA>_4_‐PFPE), were characterized by ^1^H and ^19^F NMR spectra (Figure S3–S5), Fourier‐transform infrared spectroscopy (FTIR) spectra (Figure S6) and size‐exclusion chromatography (SEC) (Table S1). Data from the ^1^H and ^19^F NMR spectra enabled us to conclude that all three types of parent polymers had been successfully synthesized with narrow molecular weight distribution (*Đ*=1.10, 1.04, and 1.04 for P<2‐HEA>_5_‐PFPE, P<OEGA>_5_‐PFPE, and P<MSEA>_4_‐PFPE, respectively, Table S1). Thus, indicating a high level of control over both the molecular weight and distribution through RAFT polymerization. It is worth noting that the RAFT polymerization was performed under mild reaction conditions, as compared with the organic synthesis used for other fluorosurfactants, which reduces the risk of side reactions and degradation of the polymer product.

The number of hydrophilic ‘head’ groups (i.e., degree of polymerization DP or composition) is a key parameter in controlling the solubility of surfactant and thus the stability of w/o droplets. Automated chromatography separation allows rapid generation of fractionated polymer libraries spanning a wide range of compositions starting from just one single parent material (Figure 1b)^[19]^ promising a rapid screening of the best composition. Thin‐layer chromatography (TLC) analysis was first conducted to optimize the solvent pair and gradient to be used for chromatography separation (Figure S7). Automated fractionation of each parent polymer was achieved in under 1 h using the optimized dichloromethane (DCM), tetrahydrofuran (THF), and methanol (MeOH) solvent gradient, a commercially available silica chromatography column, and an evaporative light‐scattering detector (Figure S8). Fractionated polymers with DP ranging from 1 to 8 were collected and characterized using ^1^H NMR (Figure S9–S11) and SEC (Table S2–S4). We next investigate the impacts of composition (or DP) on hydrophilic‐lipophilic balance (HLB), solubility in fluorinated oil, and surface tension (IFT) and ultimately the stability of droplets.

HLB value is a semi‐empirical scale for selecting surfactants based on the proportion of hydrophilic to lipophilic groups in the surfactant molecule.^[20]^ In general, HLB values between 3 and 6 stabilize w/o emulsions, while those between 8 and 18 stabilize oil‐in‐water (o/w) emulsions.^[21]^ The HLB value of both the parent and fractionated polymers were then calculated and are displayed in Table S5–S7. Accordingly, the parent P<2‐HEA>_5_‐PFPE and P<MSEA>_4_‐PFPE had a HLB value of 4.15 and 4.52, respectively, while the P<OEGA>_5_‐PFPE had a HLB value of 10.39, indicating different droplet formulations in solutions. The fractionated P(2‐HEA)_n_‐PFPE and P(MSEA)_n_‐PFPE had HLB values ranging from 1.89 to 5.87 while the P(OEGA)_n_‐PFPE polymers with DP of 3–6 had higher HLB values above 7. Therefore, we determined that parent P<2‐HEA>_5_‐PFPE and P<MSEA>_4_‐PFPE and fractions could be utilized to stabilize w/o emulsions, whereas only fractionated P(OEGA)_1_‐PFPE polymer was suitable for w/o emulsions.

**Figure 2:**
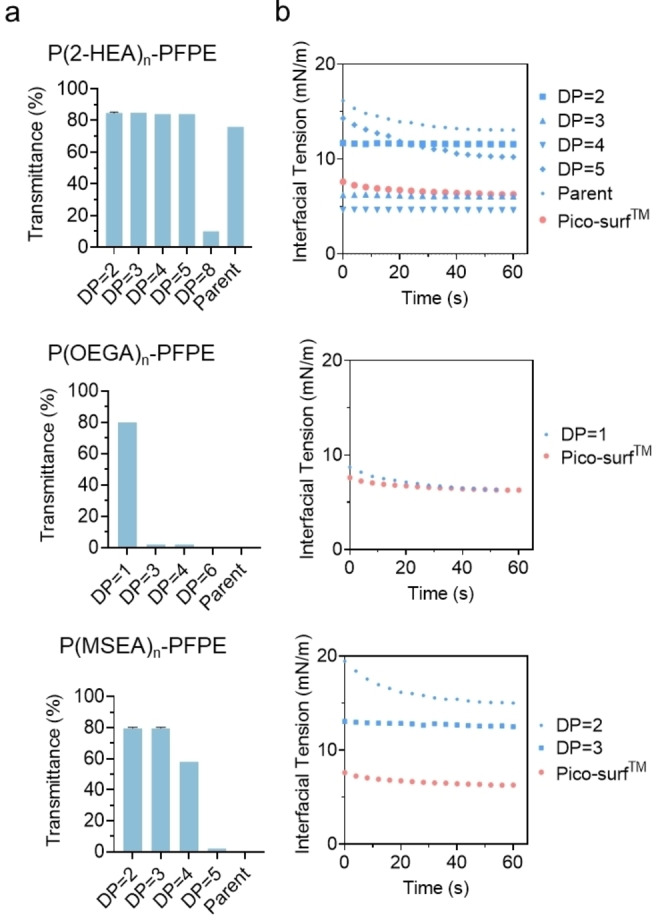
a) Transmittance of surfactant in fluorinated oil. b) Dynamic surface tension of parent and fractionated P(2‐HEA)_n_‐PFPE, P(OEGA)_n_‐PFPE, and P(MSEA)_n_‐PFPE polymers (Data are presented as mean±s.d., *n*=3).

We then evaluated the solubility of the parent polymer and its fractions by analyzing their transmittance (Figure 2a) after dissolving them in Novec^TM^ HFE7500 oil at 2 % (w/w) using Ultraviolet‐Visible (UV/Vis) spectroscopy. The results showed that polymers with high DPs exhibited limited transmittance, suggesting decreased solubility as the hydrophilic head group increased. Polymers that showed good solubility in HFE7500 oil, such as DP=2–5 P(2‐HEA)_n_‐PFPE, P(OEGA)_1_‐PFPE, and DP=2 and 3 P(MSEA)_n_‐PFPE, were tested using IFT analysis to determine the effect of the number of hydrophilic groups (Figure 2b and Table S5–S7). We found that all different surfactants show a relatively low IFT ranging from 4.60–15.00 mN/m, as compared to the commercialized product Pico‐Surf^TM^, which has a IFT of 6.28 mN/m, The DP=3 and 4 P(2‐HEA)_n_‐PFPE showed significantly smaller values. P(2‐HEA)_4_‐PFPE (4.60 mN/m), P(MSEA)_3_‐PFPE (12.48 mN/m), and P(OEGA)_1_‐PFPE (6.32 mN/m) demonstrated the lowest IFT as compared with other fractions within each type of surfactant. To ensure solubility in fluorine oil, polymer samples with high transmittance were subjected to droplet generation experiments using polydimethylsiloxane (PDMS) microfluidic devices to evaluate their ability to stabilize droplets in various solutions.

### Stability of microfluidic droplets

**Figure 3:**
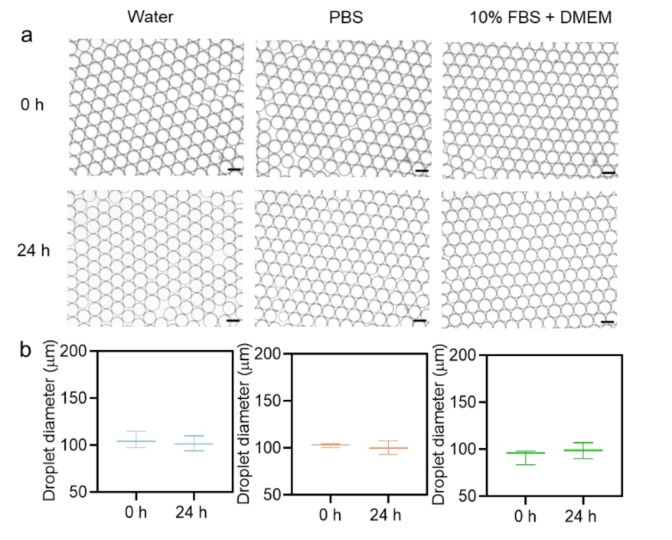
Droplet stability test of surfactant. a) Micrographs displaying the size distribution of the P(2‐HEA)_4_‐PFPE stabilized droplets, showing steady conditions during 24 h incubation at room temperature under PBS, water and DMEM+10 % FBS dispersed phase. b) Box and scatterplot of droplet size distribution during 24 h incubation at RT. The ImageJ line profiling tool was applied to measure 100 droplets to determine the mean average droplet diameter value. Scale bar, 100 μm.

To evaluate the performance of surfactants with different head groups (i.e., P(2‐HEA), P(OEGA), and P(MSEA)), w/o emulsions were generated on a microfluidic chip (Figure S12) using a 2 % (w/w) of surfactant dissolved in HFE7500 oil as the continuous phase. Droplet sizes around 100 μm were generated with different solutions including water, PBS, and cell culture medium (DMEM+10 % FBS), and the commercialized surfactant Pico‐Surf™ (2 % (w/w) in Novec™ HFE7500) was used as a reference standard (Figure S13). Polymers with satisfied transmittance were used for the generation of droplets to investigate the influence of hydrophilic head groups on droplet stability. As shown in Figure S14–S16, parent P<2‐HEA>_5_‐PFPE polymer was able to stabilize droplets in water and PBS, however the stability of cell culture medium emulsions after 24 h is relatively poor as compared with the fractionated P(2‐HEA)_4_‐PFPE. Among all fractionated polymers, P(2‐HEA)_4_‐PFPE polymers show the best performance with excellent stability in all three different solutions (Figure 3), with no significant coalescence or phase separation after one month storage at room temperature (Figure S17). In contrast, the P(OEGA)_1_‐PFPE polymer‐based emulsions were demonstrated with poor stabilities in PBS solution (Figure S18). Though the parent P<MSEA>_4_‐PFPE polymers were not dissolved well in HFE7500 oil, the fractionated polymers with DP=2 and 3 show excellent performance in stabilization of emulsions within all three different solutions (Figure S19–S21).

Generally, emulsion systems with high interfacial tensions tend to coalesce or separate out more easily due to a reduction in interfacial area, that is driven by higher interfacial energy.^[22]^ Among all different types of PFPE polymers, both P(2‐HEA)_4_‐PFPE and P(MSEA)_3_‐PFPE showed the lowest interfacial tension (Tables S5, S7), making them ideal candidates for generating w/o emulsion droplets. It is worth noting that the parent polymers with head group numbers matching those of the fractionated polymers having low interfacial tension and good performance showed satisfactory ability in stabilizing water and PBS emulsions. However, for their use in cell culture medium, a more precisely controlled molecular weight distribution is required, as evidenced by the superior stability of P(2‐HEA)_4_‐PFPE in cell culture medium (Figure 3). We anticipate that the chromategraphy purification process is highly beneficial in obtaining surfactants suitable for conditions that require a high degree of purity.

**Figure 4:**
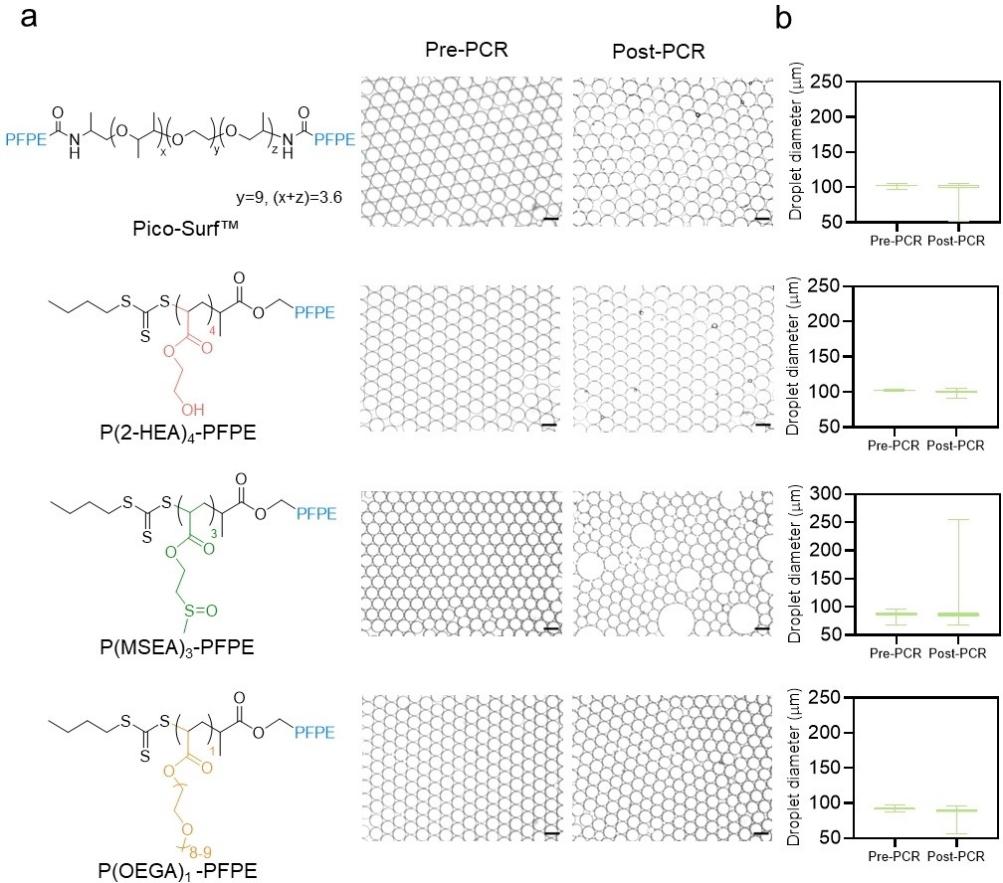
Thermostability of the droplets pre‐PCR and post‐PCR. a) Micrographs displaying the size distribution of the P(2‐HEA)_4_‐PFPE, P(MSEA)_3_‐PFPE, P(OEGA)_1_‐PFPE, and Pico‐Surf™ stabilized droplets before PCR cycles. Pico‐Surf™, P(2‐HEA)_4_‐PFPE, and P(OEGA)_1_‐PFPE stabilized droplets exhibited practically negligible merging after 35 cycles of PCR. Only P(MSEA)_3_‐PFPE slightly merged after the PCR cycles. b) Box and scatterplot of droplet size distribution during PCR. The ImageJ line profiling tool was applied to measure 100 droplets to determine the mean average droplet diameter value. Scale bar, 100 μm.

Microfluidics is a versatile technology that enables accurate and swift handling of minute fluid volumes, making it an appealing choice when combined with PCR—a process used for the fast replication of a specific DNA segment in large copy numbers.^[23]^ For droplet PCR, the emulsion droplets must remain stable during numerous thermal cycles, which expose them repeatedly to high temperatures of approximately 95 °C. Therefore, in addition to the stability of emulsion droplets in different solutions, we further investigated their stability during PCR cycles (Figure 4). Following the PCR reaction of 35 cycles at temperatures ranging from 60 to 98 °C, it was observed that droplets stabilized by P(2‐HEA)_4_‐PFPE (IFT=4.60 mN/m) showed almost no coalescence in comparison to the parent and other fractionated polymers (e.g., DP=2, 3, and 5) (Figure S22). This could be attributed to the low IFT of diblock copolymer and high level of purity, as documented in previous studies.^[10]^ Similar to the P(2‐HEA)_4_‐PFPE, droplet stabilized by P(OEGA)_1_‐PFPE (IFT=6.32 mN/m) also demonstrated minimal coalescence after PCR cycles (Figure 4a). It is worth noting that both surfactants performed better than the commercial Pico‐Surf™ surfactant, as shown in Figure 4, which can be attributed to the synthesis limitations of the tri‐block co‐polymer surfactants.^[10]^ However, P(MSEA)_n_‐PFPE with DP=2 and 3 was not able to stabilize droplets after PCR cycles (Figure S23). The ability of all four types of surfactants to stabilize droplets was demonstrated by a quantitative analysis of the post‐PCR droplet size distributions (Figure 4b). The Bancroft rule states that water‐soluble surfactants tend to stabilize o/w emulsions.^[24]^ A surfactant's solubility and emulsification type can be predicted based on its hydrophilic‐lipophilic balance (HLB) value.^[25]^ The HLB values of the DP=3–6 P(OEGA)_n_‐PFPE ranged from 7 to 11 (Table S6), which is more suitable for an o/w emulsifier. Consequently, DP=3–6 P(OEGA)_n_‐PFPE showed complete merging of droplets after PCR.

### Inter‐droplet transfer

**Figure 5:**
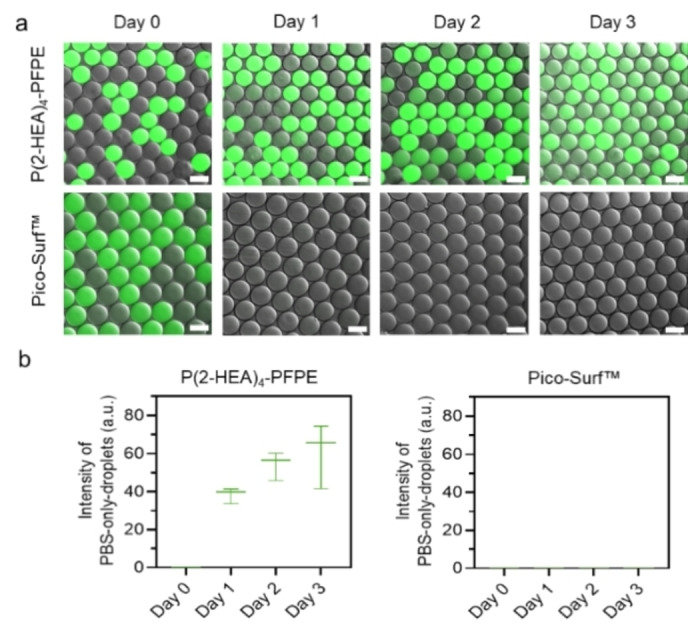
Dye diffusion test of surfactant. a) Micrographs of inter‐droplet diffusion of fluorescein salt after 3 days of stabilization with P(2‐HEA)_4_‐PFPE or Pico‐Surf™ of groups of empty and dye‐containing droplets. b) Box‐plot of PBS‐only‐droplets fluorescence intensity during 72 h incubation at 37 °C. The ImageJ line profiling tool was applied to measure 5 PBS‐only‐droplets to quantify the fluorescence intensity (Data are presented as mean±s.d., *n*=5). Scale bar, 100 μm.

Although surfactants are employed, droplets may not function as entirely sealed containers, as small molecules can still permeate the surfactant layer and enter the oil phase. Therefore, we studied the effectiveness of our surfactant in preventing inter‐droplet molecular transport by using a small water‐soluble dye, sodium fluorescein salt. We collected equal amounts of PBS‐only and PBS+fluorescein dye droplets, which were generated and collected with Eppendorf tubes and then incubated at 37 °C for up to 3 days. As shown in Figure 5, the fluorescence signal of Pico‐Surf™ stabilized droplets remain zero at day 1, 2, and 3, indicating the diffusion of fluorescence dyes into the outer phase of droplets. Furthermore, analysis of inter‐droplet transport kinetics using the 3 μM sodium fluorescein salt revealed that for these two surfactants, less leakage occurred on day 1 compared to the leakage that took place on day 3. Droplets prepared with the P(2‐HEA)_4_‐PFPE surfactant were more resistant to dye diffusion as compared to those created by the commercial Pico‐Surf™ (2 % (w/w) in Novec^TM^ HFE7500) surfactant, indicating that the brushed structured headgroup improves dye‐retention and therefore reduces inter‐droplet transfer. Notably, there was no significant difference in the surface tension values measured for both fluorosurfactants when we tested their interfacial tension.

### Double emulsion for yeast cell culture

In recent years, advances in droplet microfluidics have paved the way for the analysis of individual cells encapsulated within picoliter‐sized microdroplets.^[4c]^ In this context, the use of water‐in‐oil‐in‐water (w/o/w) double emulsion (DE) has become common in droplet‐based single‐cell analysis. This is mainly because the outer water phase is compatible with established commercial analytical methods, such as flow cytometry.[^6c^, ^9a^, ^9b^] A DE is a complex soft colloidal system with a core–shell structure, consisting of an immiscible oil phase that separates an aqueous core or multiple cores from the outer carrier aqueous phase.^[26]^ This system is stabilized by two sets of surfactants, with surfactants in the oil phase and outer water phase stabilizing the interface of the water‐in‐oil internal emulsion and oil‐in‐water emulsion, respectively. DEs are considered to be metastable systems with two protective shells that provide protection against oxidation, degradation, and corrosion.^[27]^ Despite this protection, DEs are thermodynamically unstable and can rupture during storage.^[26b]^

**Figure 6:**
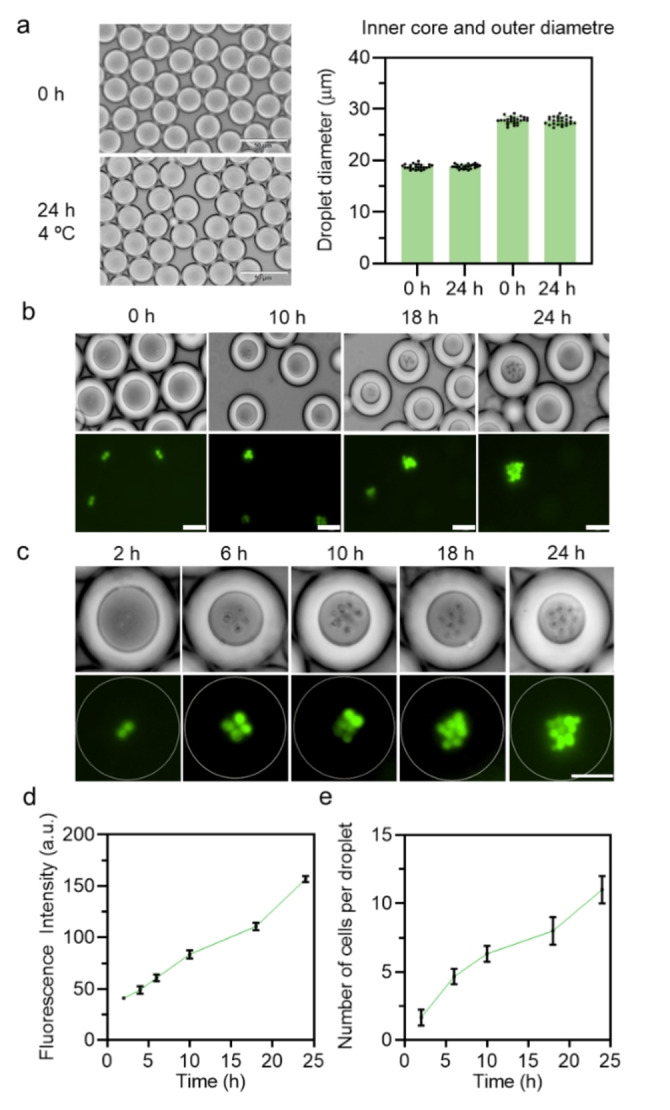
DE system to test surfactant biocompatibility. a) Micrographs displaying the size distribution of the P(2‐HEA)_4_‐PFPE stabilized w/o/w double emulsion, showing steady conditions during 24 h incubation at 4 °C under yeast cell medium dispersed phase. Scale bar, 50 μm. b) Micrographs displaying the bright field and fluorescence of yeast cells within double emulsion at 4 time points. Scale bar, 30 μm. c) Micrographs displaying the fluorescence of yeast cells per droplet at 5 time points. The ImageJ line profiling tool was applied to quantify the fluorescence intensity. Scale bar, 50 μm. d) Box‐plot shows yeast cells fluorescence intensity during 24 h incubation at 30 °C. e) Box‐plot shows number of yeast cells per droplet during 24 h incubation at 30 °C. (Data are presented as mean±s.d., *n*=3)

Fluorocarbon surfactants, such as commercial options like 008‐FluoroSurfactant (RAN Biotechnologies) and PicoSurf^TM^ (Sphere Fluidics), are commonly used for the generation of DEs.^[28]^ Consequently, we have assessed the performance of our fluorosurfactants in terms of their stability and biocompatibility for yeast cell culturing. Using yeast cell medium disperse as inner phase, 3 % (w/v) P(2‐HEA)_4_‐PFPE were used to generate droplets with a diameter of ≈30 μm (Figure S24 and Figure 6a). After 24 h incubation, the P(2‐HEA)_4_‐PFPE‐stabilized DE droplets exhibited excellent stability under 4 °C, with the diameter of the inner core and outer shell remaining constant at approximately 20 μm and 30 μm, respectively. This finding suggests that P(2‐HEA)_4_‐PFPE has potential in stabilizing double emulsions for yeast cell culture.

Subsequently, we tested the growth of a GFP‐tagged *S. cerevisiae* strain (GEN.PK2‐1C) in water‐in‐oil‐in‐water (w/o/w) DEs, which can overcome the poor stability of single emulsions such as droplet shrinkage.^[29]^ The number of fluorescent yeast cells per droplet was observed to increase over time, as shown in Figure 6b. We measured the total fluorescence intensity of yeast cells per droplet at 6 time points (2, 4, 6, 10, 18, and 24 h) and the number of yeast cells per droplet by comparing the bright field and fluorescence images (Figure 6c). After 24 h incubation (50 rpm, 30 °C), the number of yeast cells per droplet increased from 2 to 12, while the average fluorescence intensity of cells in the droplets had increased to 3 times more than the original state (Figure 6d, e).

We further assessed the biocompatibility of the new surfactant in single emulsions. αIIbβ3‐expressing Chinese hamster ovary (CHO) cells (A5 cells) were released from droplets using 1H,1H,2H,2H‐perfluoro‐1‐octanol (PFO) and a viability assay conducted using Trypan Blue solution. A5 cells exhibited an 80–90 % survival rate at each time point (Figure S25). In comparison, cells cultured in traditional wells showed approximately 95 % viability. These results indicate that P(2‐HEA)_4_‐PFPE is non‐toxic to cells and suitable environment for cell‐based experiments.

## Conclusion

In conclusion, this study introduces a novel and efficient method utilizing LRP to synthesize tailor‐made fluorosurfactants for achieving highly stable emulsions in droplet‐based microfluidics. This approach offers numerous advantages, including cost‐effectiveness, versatility, and precise control over surfactant properties, poised to revolutionize the field of droplet‐based microfluidics. Through the optimization of hydrophilic head groups, we have demonstrated that our fluorosurfactants exhibit superior performance, providing ultra‐high droplet stabilization across a variety of biological environments and even under high‐temperature conditions. Additionally, we have shown a significant reduction in the inter‐droplet transfer of small molecules compared to conventional multiblock fluorosurfactants. Furthermore, we have successfully applied our surfactants in the creation of double emulsions for yeast cell culturing, which may in the future help to better automate current in vitro model systems of disease.[^26a^, ^30^] We anticipate that the LRP‐based polymerization strategy, coupled with our customized fluorosurfactants, will pave the way for significant advancements in the next generation of tailored fluorosurfactants, facilitating a wide range of applications in the field of cutting‐edge droplet‐based microfluidic technology.

## Conflict of interest

The authors declare no conflict of interest.

1

## Supporting information

As a service to our authors and readers, this journal provides supporting information supplied by the authors. Such materials are peer reviewed and may be re‐organized for online delivery, but are not copy‐edited or typeset. Technical support issues arising from supporting information (other than missing files) should be addressed to the authors.

Supporting Information

## Data Availability

The data that support the findings of this study are available from the corresponding author upon reasonable request.
