# Supplementary material for: Tailored Fluorosurfactants through Controlled/Living Radical Polymerization for Highly Stable Microfluidic Droplet Generation
Source: Angew Chem Int Ed Engl. 2023 Dec 12;63(3):e202315552. doi: 10.1002/anie.202315552 (PMC10952479; doi:10.1002/anie.202315552)
Supplement: Supplementary file 1 — Supporting Information [file ANIE-63-0-s001.pdf]

Angewandte Chemie
Eine Zeitschrift der Gesellschaft Deutscher Chemiker

Supporting Information

Tailored Fluorosurfactants through Controlled/Living Radical Polymerization for Highly Stable Microfluidic Droplet Generation

X. Li, S.-Y. Tang, Y. Zhang, J. Zhu, H. Forgham, C.-X. Zhao, C. Zhang, T. P. Davis*, R. Qiao**

SYNTHETIC AND CHARACTERIZATION METHODS

Materials. The hydroxy-terminated perfluorinated poly (propylene ether) (PFPE-OH, Mw ~ 2000 g/mol,) was purchased from The Chemours Company. Translucent K^+ Free Yeast Nitrogen Base (YNB) was purchased from Formedium Ltd. Gibco™ L-glutamine and Gibco™ MEM Non-Essential Amino Acids Solution (NEAA) were purchased from Thermo Fisher Scientific Inc. The Milli-Q water (~18.2 MΩ·cm^{-1}) was from Milli-Q® Direct systems. The HFE7500 oil was purchased from 3 M (St. Paul, MN, USA). 2-Hydroxyethyl acrylate (96%), acryloyl chloride (97%), Poly (ethylene glycol) methyl ether acrylate (OEGA, M_w = 480 g/mol), 1H,1H,2H,2H-perfluoro-1-octanol (PFO), Dulbecco′s Modified Eagle′s Medium (DMEM), Fetal Bovine Serum (FBS), Dulbecco′s Phosphate Buffered Saline (DPBS), glucose, potassium chloride, Pluronic F-127, Tween 20, OptiPrep™ Density Gradient Medium, Fluorescein sodium salt, Trypan Blue solution, N-(3-(dimethylamino)propyl)-N′-ethylcarbodiimide hydrochloride (EDCl), 4-(dimethylamino)pyridine (DMAP), α,α,α-Trifluorotoluene (TFT), Dichloromethane (DCM), Tetrahydrofuran (THF), Methanol (MeOH), and 2,2′-azobis(2-methylpropionitrile) (AIBN) were purchased from Sigma-Aldrich and used as received. The synthesis of 2-(Methylsulfinyl)ethyl Acrylate was prepared according to previous literature.[1] The RAFT agent 2-(n-Butyltrithiocarbonate)-propionic acid (BTPA) was prepared according to previous literature.[2]

Synthesis of RAFT polymerization surfactants. RAFT polymerisation was used to produce a PFPE-<2-HEA, OEGA, MSEA>-based surfactant with 5 degrees of polymerisation. In a typical experiment, the BTPA–PFPE macro-RAFT agent (400 mg, 0.18 mmol), AIBN (6.0 mg, 0.036 mmol) and three kinds of monomers 2-HEA (104.6 mg, 0.90 mmol), OEGA (1.5 g, 3.13 mmol), MSEA (146.1 mg, 0.90 mmol) were dissolved in TFT (2 ml) in three flask glasses respectively. Subsequently, argon deoxygenated the mixed solution for 15 minutes, followed by 4 h reactions in a 70 °C oil bath. Finally, P<2-HEA>$_5$-PFPE, P<OEGA>$_5$-PFPE and P<MSEA>$_4$-PFPE were dried at 60 °C under vacuum to get a yellow viscous liquid.

Nuclear Magnetic Resonance (NMR). ^{1}H NMR spectra of polymer solutions in deuterated chloroform ($CDCl_3$) were acquired on a Bruker AVANCE 400 MHz spectrometer at 25 °C. A 90° pulse width 14 µs, relaxation delay 1 s, acquisition time 4.1 s, and 64 scans were used in all measurements. ^{19}F NMR spectra were acquired on a Bruker AVANCE 400 MHz spectrometer at 25 °C. The solvent was $CDCl_3$. A 90° pulse width 14 µs, relaxation delay 1 s, acquisition time 4.1 s, and 128 scans were used in all measurements.

Size Exclusion Chromatography (SEC). SEC was used to determine molecular weights and molecular weight distributions using a Waters Alliance 2690 separations module outfitted with a Waters 2414 refractive index (RI) detector, a Waters 2489 UV/Vis detector, a Waters 717 Plus autosampler, and a Waters 1515 isocratic HPLC pump. The mobile phase was THF at a flow rate of 1 mL/min. Before testing, the samples were dissolved in THF at a predetermined concentration (1 mg/mL) and passed through 0.45 µm PTFE filters. The molecular weight was determined in comparison to polystyrene standards.

Flash Chromatography. Automatic flash chromatography was carried out using a compact Pure Chromatography Systems (BUCHI, Australia) equipped with an ELSD and UV detection, utilizing FlashPure Silica cartridge (80 g, 40 µm irregular particle shape; BUCHI) eluted with appropriate solvent pairings. All the chromatographic solvents were ACS grade or above and were utilized without additional purification.

Cell preparation. αIIbβ3-expressing Chinese hamster ovary (CHO) cells (A5 cells) were cultured in DMEM supplemented with 10% FBS, 1% L-Glutamine, 1% NEAA, 0.35% Geneticin. GFP-tagged S. cerevisiae strain (CEN.PK2-1C-GFP) was used in cell growth assays. We pre-cultured CEN.PK2-1C-GFP overnight at 30 °C, 50 rpm in synthetic drop-out medium[3] and washed cell twice and re-inoculated into 5ml uracil drop-out medium containing defined concentrations of potassium (1 × translucent K+ Free YNB, 1% glucose, and 0, 1, 10, and 50 mM potassium chloride).

Chip fabrication. The flow-focusing water-oil (single) or water-oil-water (double) emulsion droplet generator used in this

study was fabricated by standard photolithography technique. A designed channel was patterned to silicon wafer with SU-8 2100 or 2035 (MicroChem, Germany) to obtain a mold. Then, polydimethylsiloxane (PDMS) in liquid form is prepared by mixing the based and curing agent (10:1 in weight, respectively) to duplicate the pattern on the mold. After a plasma cleaning process on the surface of glass slide and PDMS, the surface energy would be increased and finally pressured to each other for an irreversible bonding.

Single and double emulsion generation. To generate stable water-oil single droplets and make sure good encapsulation of cells, disperse phase here was one of water, PBS and DMEM + 10% FBS, and 2% (w/w) in Novec^{TM} HFE7500 (3M, St. Paul, MN, USA) fluorosurfactant was used as continue phase. Two syringe pumps (Harvard Apparatus, USA) were used to inject the dispersed phase and continue phase respectively. We set the flow rates of two phases as 1:3 (dispersed phase: 200 µl/h; continue phase: 600 µl/h).

To generate stable double emulsion (DE) droplets and make sure good encapsulation of cells, disperse (inner) phase here was yeast cell medium added with 30% OptiPrep^{TM}, 3% (w/v) P(2-HEA)$_4$-PFPE added Novec^{TM} HFE7500 oil as middle phase, and 1% (w/v) Pluronic F-127 and 1% (v/v) Tween 20-added PBS as an outer phase. Three syringe pumps (Harvard Apparatus, USA) were used to inject the inner phase, oil, and outer phase, respectively. We set the flow rates of three phases as 1:3:10 (inner phase: 60 µl/h; oil: 180 µl/h; outer phase: 600 µl/h). A PTFE tubing (Cole-Parmer, Illinois, USA) with inner diameter 0.06 inch, outer diameter 0.02 inch was used to transfer DE microdroplets into a 2 ml Eppendorf^{TM} safe-lock tube for storage.

After yeast cells encapsulation, the DE droplets flowed into centrifugal tube (500 µl) through fluorinated ethylene propylene (FEP) tubing, and the tube was fixed on a shaker incubator at 30 °C and 50 rpm. Then, we transferred about 10 µl DE droplets to the glass slides using pipette, the fluorescence microscope was employed to measure the total fluorescence intensity of yeast cells per droplet at 2, 6, 10, 18, and 24 h, respectively.

Image acquisition and data processing. A Nikon optical microscope (Eclipse TS100 Inverted Microscope, Nikon Inc., Japan) equipped with a digital camera (Canon, Tokyo, Japan) was used to capture the droplets and images. The number of yeast cell clusters in the droplets would be easier counted by comparing the fluorescence images to bright filed images. The size distribution and average fluorescence intensity of the micro droplets was analyzed by ImageJ (National Institute of Health, USA).

Transmittance method. Ultraviolet-visible (UV-Vis) double beam spectrophotometer (UV-2450, Shimadzu, Japan) was used to measure the transmittance. The absorbance of surfactant (2% (w/w) in Novec^{TM} HFE7500) was determined at 430 nm, using quartz cells of 1.00 cm of optical path.

Interfacial tension. The interfacial tension for surfactant enriched o/w interfaces were measured by a pendant drop method using a DSA-10 drop-shape analysis unit (Krüss GmbH, Hamburg, Germany).

Fourier-transform infrared spectroscopy (FTIR). FTIR spectra were recorded on a Thermo Scientific Nicolet 5700 FTIR spectrometer equipped with a smart orbit diamond ATR unit. The wavenumber range was 4000-400 cm^{-1} and the resolution was 4 cm^{-1}. The number of scans was set as 64.

PCR experiment. Microfluidic drop making devices are used to create ~120 µm diameter monodisperse droplets stabilized by the indicated surfactants 2% (w/w) in Novec^{TM} HFE7500. Each emulsion of droplets is created from 40 µl PCR mix comprising 32 µl water, 8 µl 5x Phusion HF detergent-free Buffer (F-520L, Thermo Fisher). The PCR program is 98 °C for 30 s; then 35 cycles of 98 °C for 7 s, 60 °C for 30 s, and 72 °C for 20 s; then a final step of 72 °C for 10 min.

Dye diffusion experiment. Syringe pumps (Harvard Apparatus, USA) are used to regulate the flow of various liquid streams. For imaging, emulsion droplets were pulled by capillary force into a hollow rectangular capillary tube (dimensions: ID 0.2 x 2.0 x 50 mm; source ProSciTech Pty Ltd, Australia) and then the open ends were sealed with Vaseline grease before being affixed to microscope cover glass (thickness: 0.13-0.16 mm). At days 0, 1, 2, and 3, Zeiss

LSM 710 (Germany) was used to produce bright field and fluorescence images of fluorescein sodium salt. For the recording of green fluorescence signals, a 488 nm excitation wavelength and a 520/55 nm band pass filter were used. ImageJ was implemented to determine the fluorescence intensity of PBS-only droplets (National Institute of Health, USA).

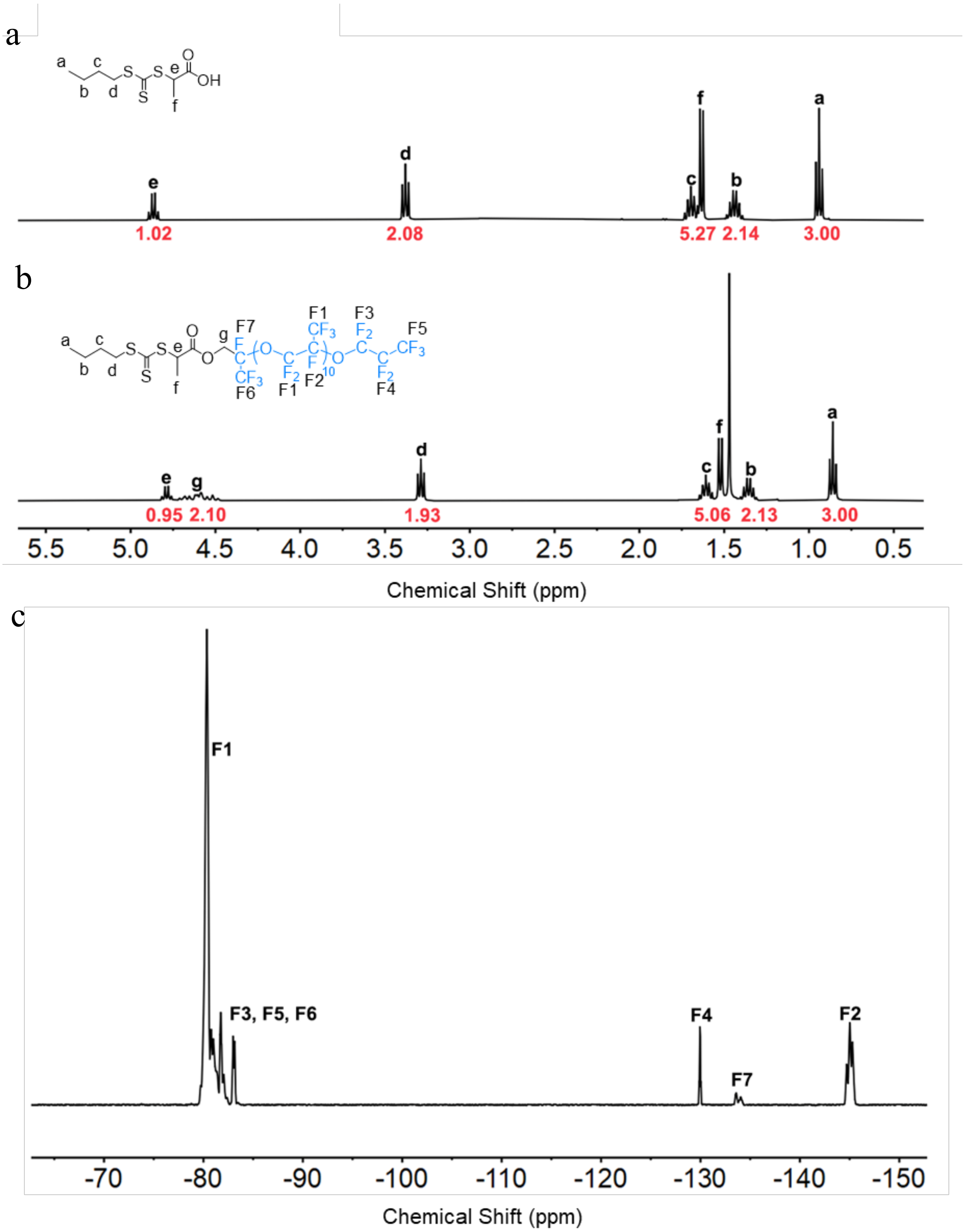

Figure S1. ^{1}H NMR spectra of 2-(butylthiocarbonothioylthio)-propionic acid (BTPA) (a) and BTPA-perfluoropolyether (PFPE) macro-CTA (b) in $CDCl_3$. (c) ^{19}F NMR spectra of BTPA-PFPE in $CDCl_3$.

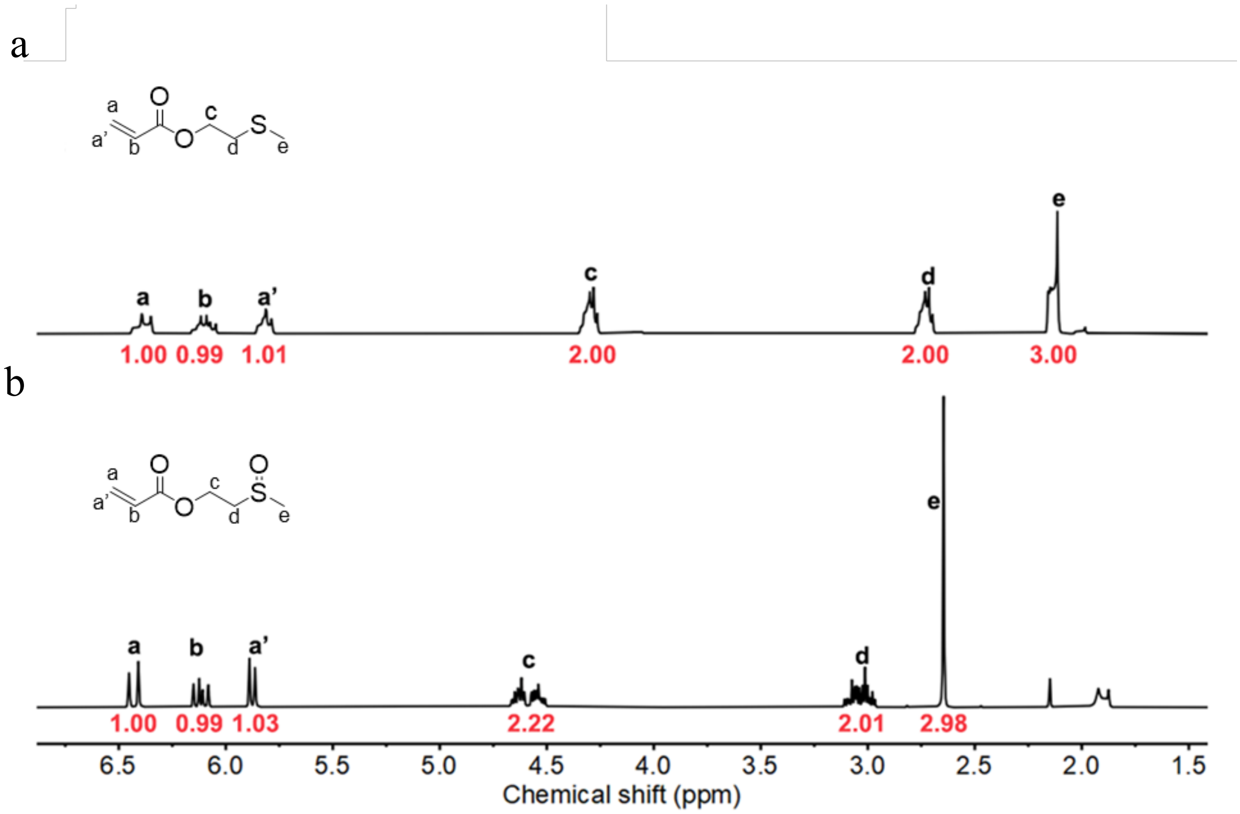

Figure S2. ^{1}H NMR spectra of 2-(methylthio)ethyl Acrylate (MTEA) (a) and 2-(methylsulfinyl)ethyl acrylate (MSEA) (b) in $CDCl_3$.

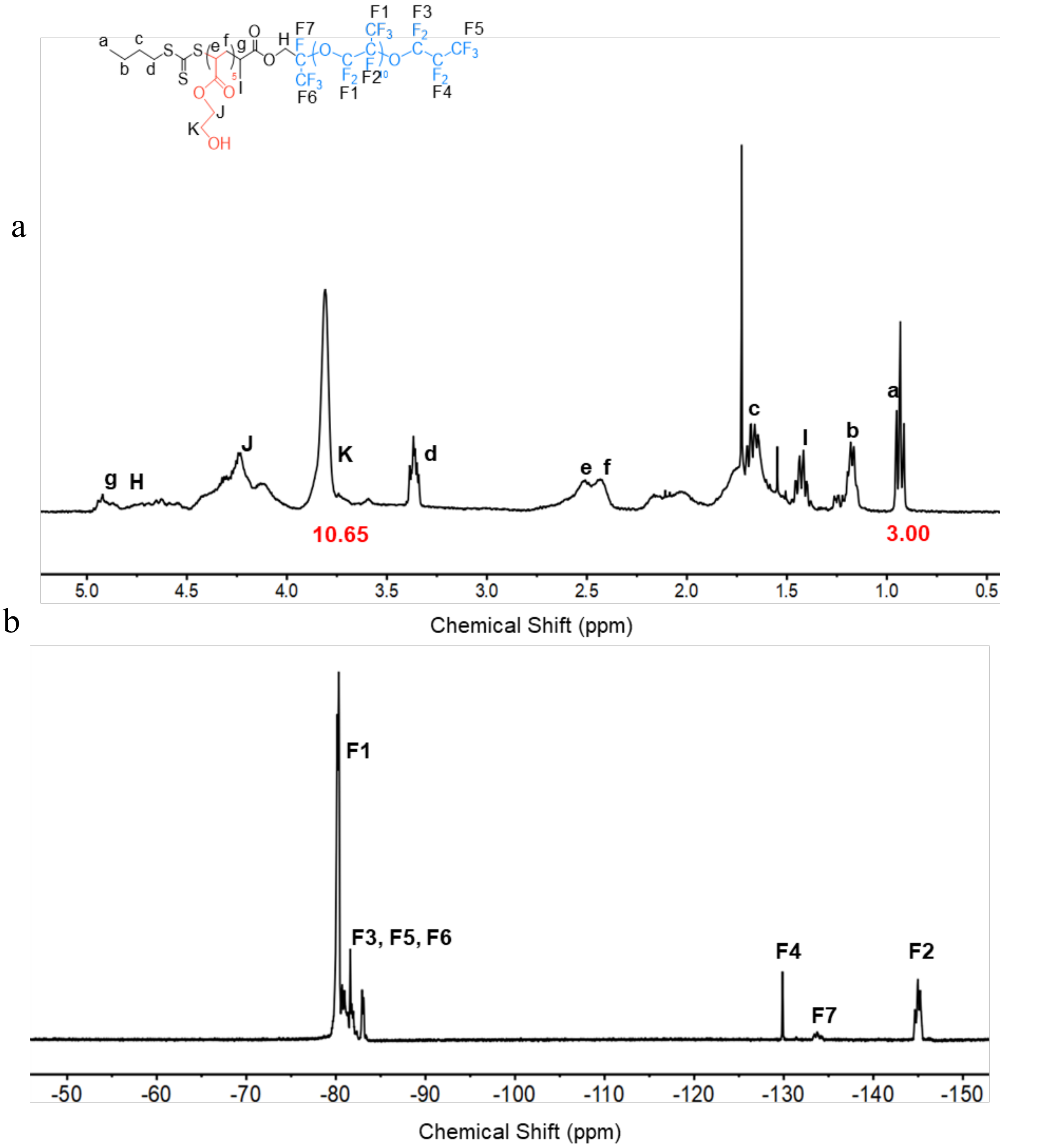

Figure S3. (a) ^{1}H NMR spectra of parent P<2-HEA>$_5$-PFPE in $CDCl_3$. (b) ^{19}F NMR spectra of parent P<2-HEA>$_5$-PFPE in $CDCl_3$.

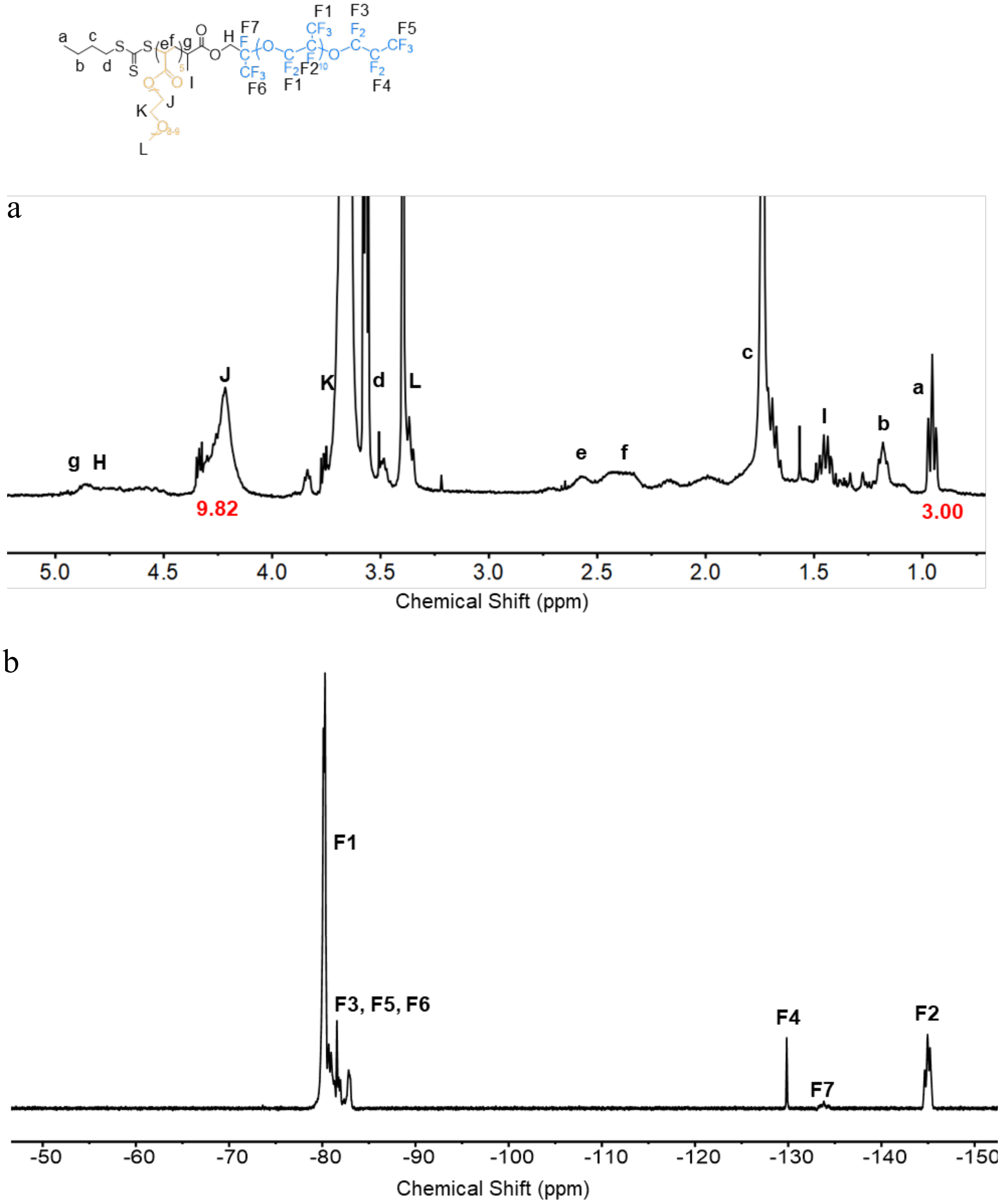

Figure S4. (a) ^{1}H NMR spectra of parent P<OEGA>$_5$-PFPE in $CDCl_3$. (b) ^{19}F NMR spectra of parent P<OEGA>$_5$-PFPE in $CDCl_3$.

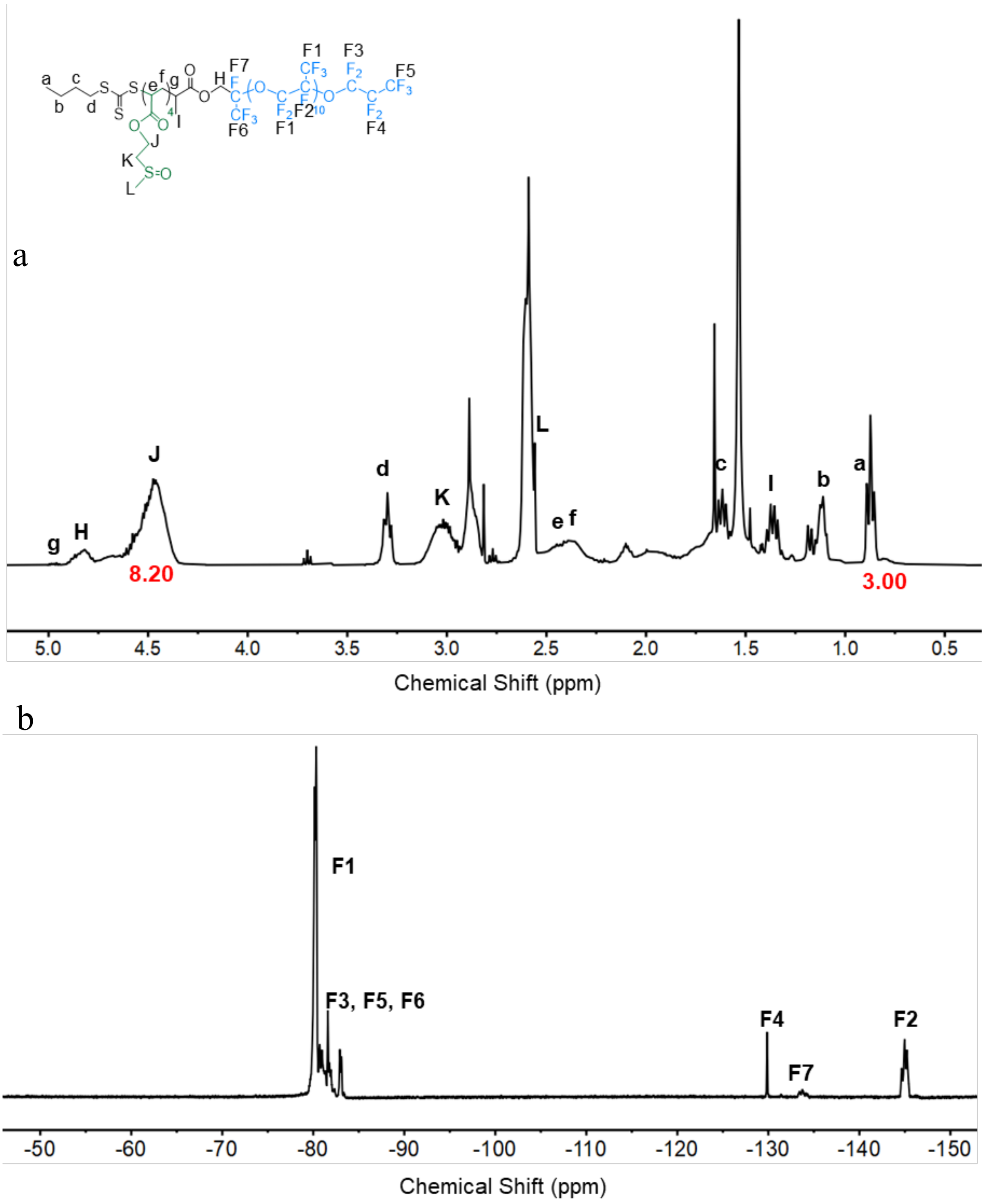

Figure S5. (a) ^{1}H NMR spectra of parent P<MSEA>$_4$-PFPE in $CDCl_3$. (b) ^{19}F NMR spectra of parent P<MSEA>$_4$-PFPE in $CDCl_3$.

Table S1. Molecular characterization data for parent P<2-HEA>$_5$-PFPE, P<OEGA>$_5$-PFPE, and P<MSEA>$_4$-PFPE.

polymer	M_n, NMR (g/mol)	*Đ*	F content (wt %)
P<2-HEA>$_5$-PFPE	2800	1.10	49
P<OEGA>$_5$-PFPE	4620	1.04	30
P<MSEA>$_4$-PFPE	3030	1.04	45

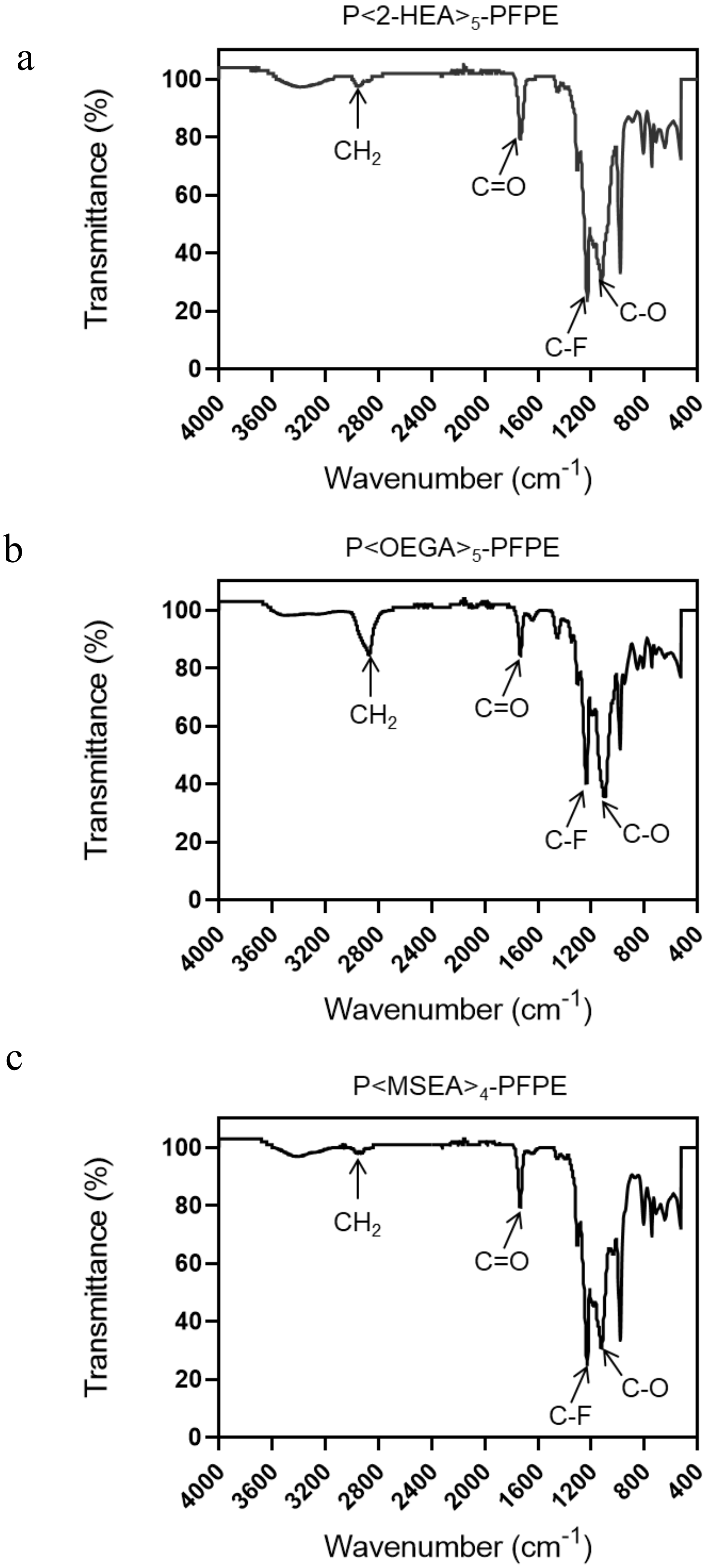

Figure S6. Characterization of synthesized surfactants. FTIR spectra of (a) parent P<2-HEA>$_5$-PFPE, (b) P<OEGA>$_5$-PFPE, and (c) P<MSEA>$_4$-PFPE.

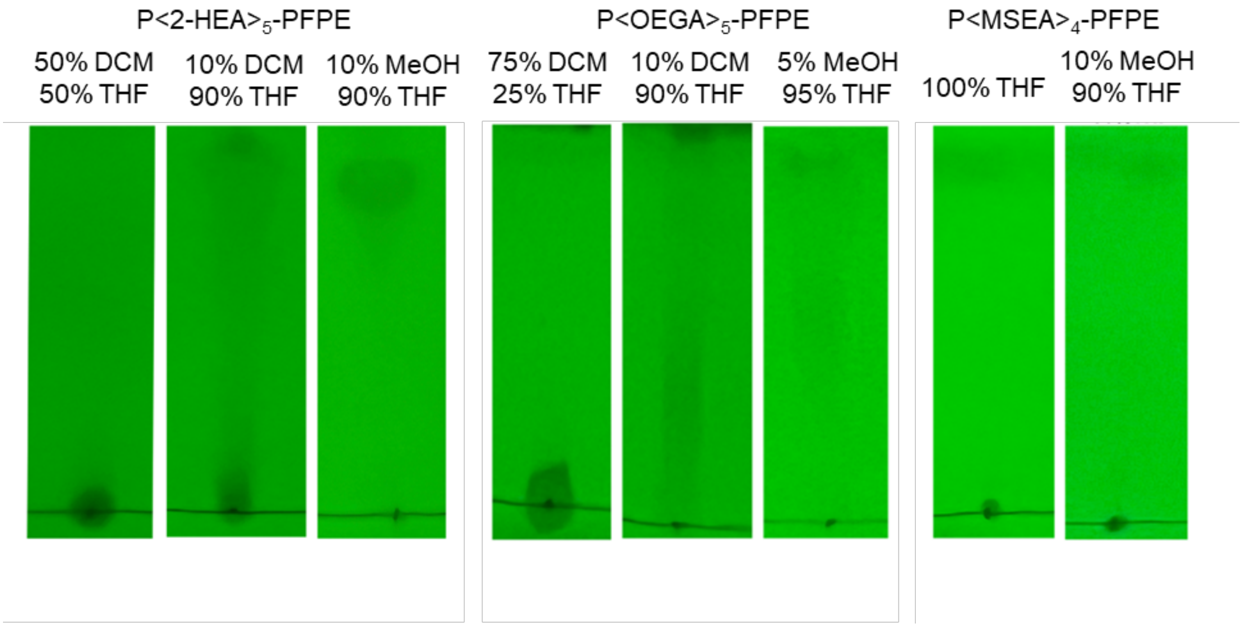

Figure S7. Thin-layer chromatography (TLC) analysis of the parent P<2-HEA>$_5$-PFPE, P<OEGA>$_5$-PFPE, and P<MSEA>$_4$-PFPE, pictured under UV light. Streaking suggests a state of the eluent that is conducive to compositional fractionation.

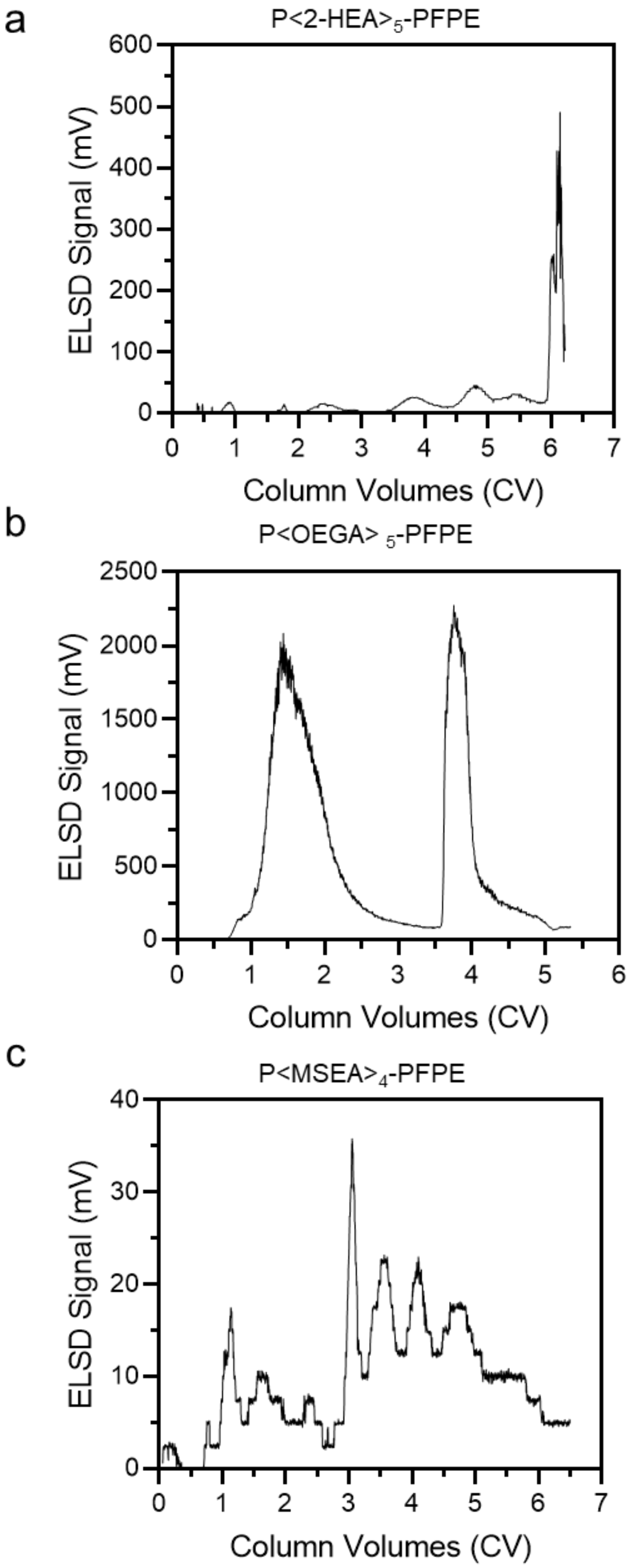

Figure S8. Representative ELSD signal for the separation of parent P<2-HEA>$_5$-PFPE, P<OEGA>$_5$-PFPE and P<MSEA>$_4$-PFPE.

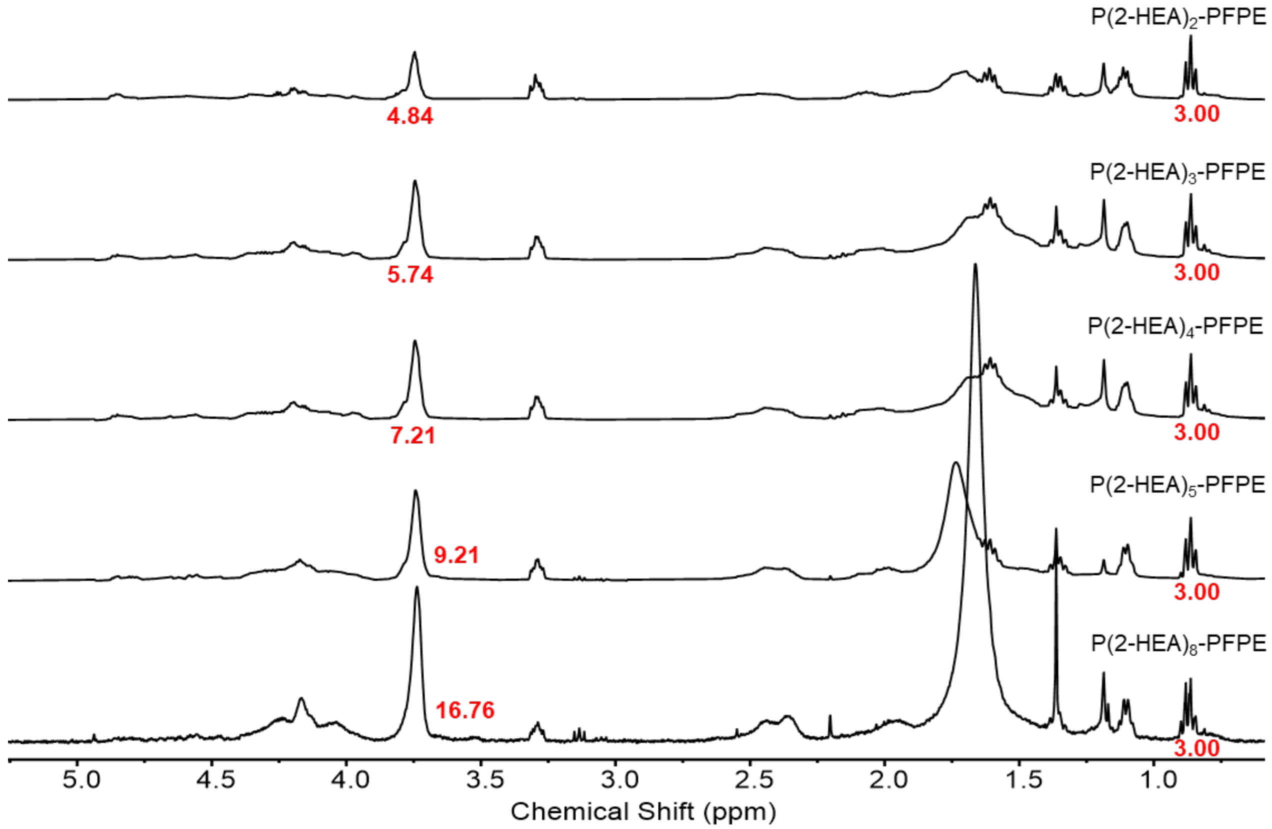

Figure S9. ^{1}H NMR spectra of fractionated P(2-HEA)$_n$-PFPE copolymers in $CDCl_3$.

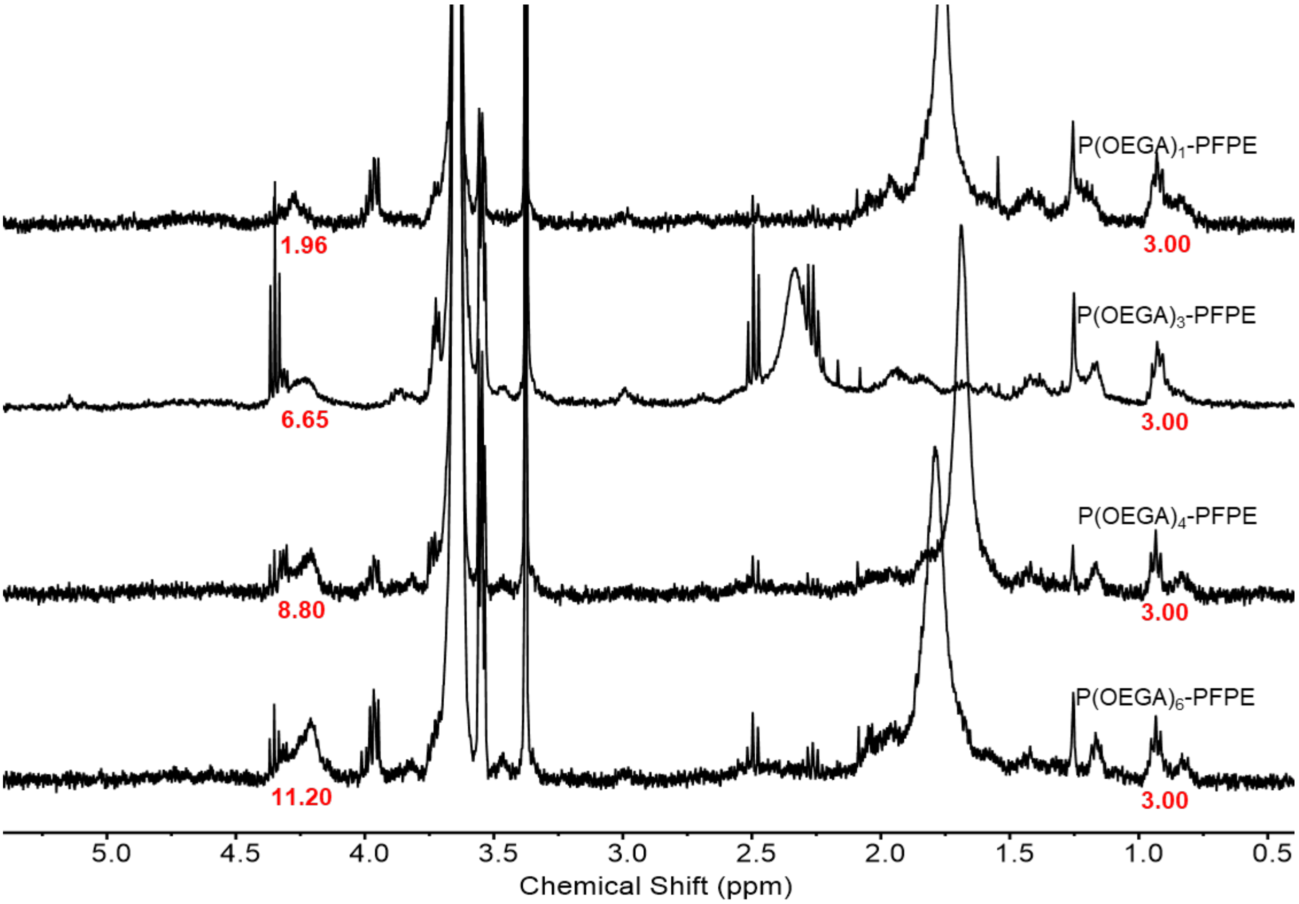

Figure S10. ^{1}H NMR spectra of fractionated $P(OEGA)_n$-PFPE copolymers in $CDCl_3$.

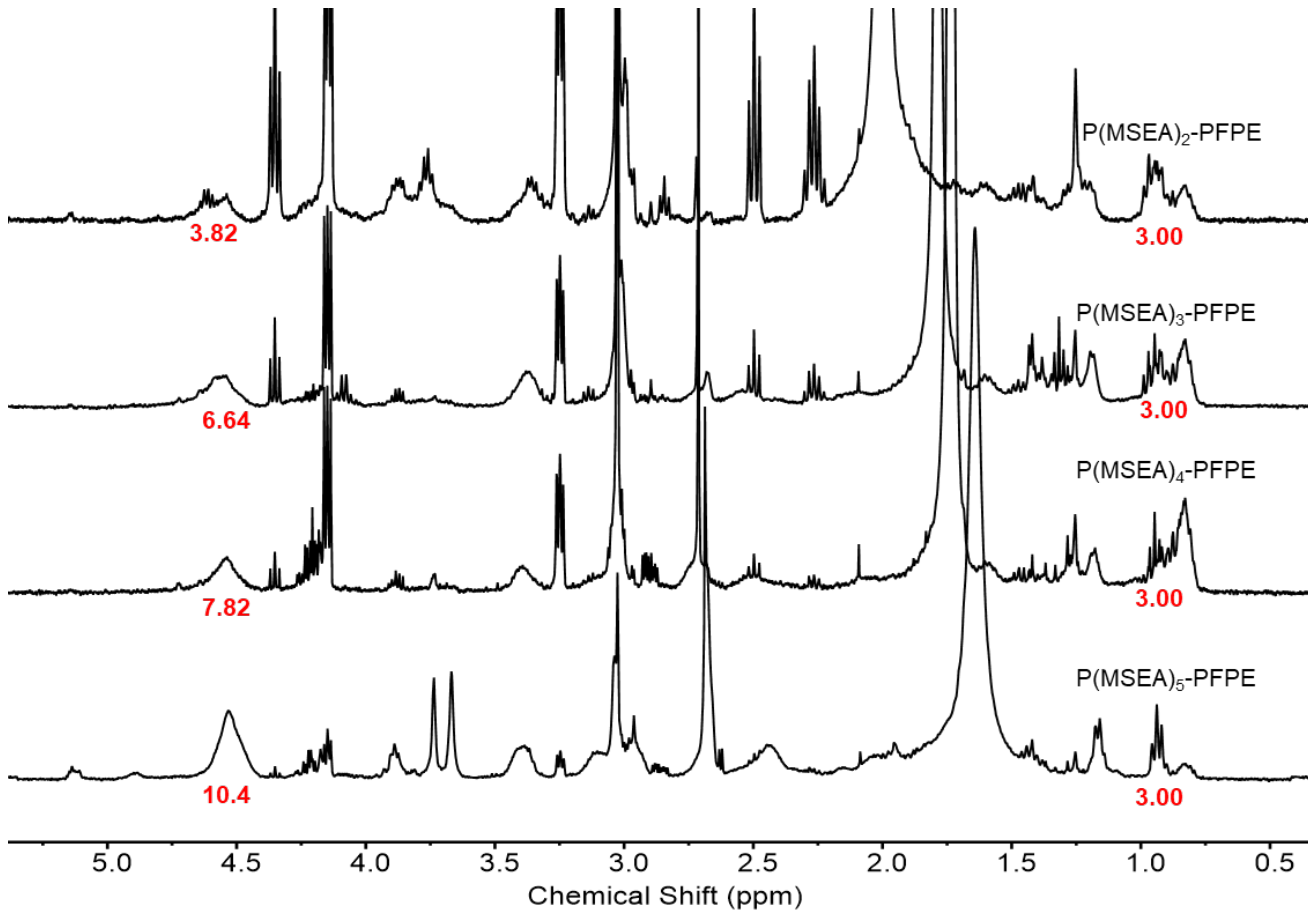

Figure S11. ^{1}H NMR spectra of fractionated $P(MSEA)_n$-PFPE copolymers in $CDCl_3$.

Table S2. Molecular characterization data for fractionated P(2-HEA)$_n$-PFPE.

polymer	M_n, NMR (g/mol)	$Đ$	F content (wt %)
P(2-HEA)$_2$-PFPE	2450	1.27	56
P(2-HEA)$_3$-PFPE	2570	1.03	53
P(2-HEA)$_4$-PFPE	2680	1.16	51
P(2-HEA)$_5$-PFPE	2800	1.15	49
P(2-HEA)$_8$-PFPE	3150	1.13	43

Table S3. Molecular characterization data for fractionated P(OEGA)$_n$-PFPE.

polymer	M_n, NMR (g/mol)	$Đ$	F content (wt %)
P(OEGA)$_1$-PFPE	2700	1.17	51
P(OEGA)$_3$-PFPE	3660	1.15	37
P(OEGA)$_4$-PFPE	4140	1.14	33
P(OEGA)$_6$-PFPE	5100	1.18	27

Table S4. Molecular characterization data for fractionated P(MSEA)$_n$-PFPE.

polymer	M_n, NMR (g/mol)	$Đ$	F content (wt %)
P(MSEA)$_2$-PFPE	2540	1.13	54
P(MSEA)$_3$-PFPE	2710	1.33	50
P(MSEA)$_4$-PFPE	2870	1.27	48
P(MSEA)$_5$-PFPE	3030	1.12	45

Table S5. Hydrophilic-lipophilic balance (HLB) value, Interfacial tension (IFT) and Transmittance of parent and fractionated P(2-HEA)$_n$-PFPE polymer.

polymer	HLB	IFT (mN/m)	Transmittance (%)
**Parent P<2-HEA>$_5$-PFPE**	4.15	13.06	76
P(2-HEA)$_2$-PFPE	1.89	11.57	85
P(2-HEA)$_3$-PFPE	2.70	6.07	85
P(2-HEA)$_4$-PFPE	3.46	4.60	84
P(2-HEA)$_5$-PFPE	4.14	10.22	84
P(2-HEA)$_8$-PFPE	5.87	N/A	10
Pico-Surf^{TM}	N/A	6.28	100

Table S6. Hydrophilic-lipophilic balance (HLB) value, Interfacial tension (IFT) and Transmittance of parent and fractionated P(OEGA)$_n$-PFPE polymer.

polymer	HLB	IFT (mN/m)	Transmittance (%)
Parent P<OEGA>$_5$-PFPE	10.39	N/A	0
P(OEGA)$_1$-PFPE	3.56	6.32	80
P(OEGA)$_3$-PFPE	7.87	N/A	2
P(OEGA)$_4$-PFPE	9.28	N/A	2
P(OEGA)$_6$-PFPE	11.29	N/A	0

Table S7. Hydrophilic-lipophilic balance (HLB) value, Interfacial tension (IFT) and Transmittance of parent and fractionated P(MSEA)$_n$-PFPE polymer.

polymer	HLB	IFT (mN/m)	Transmittance (%)
Parent P<MSEA>$_4$-PFPE	4.52	N/A	0
P(MSEA)$_2$-PFPE	2.55	15.00	80
P(MSEA)$_3$-PFPE	3.60	12.48	80
P(MSEA)$_4$-PFPE	4.52	N/A	58
P(MSEA)$_5$-PFPE	5.35	N/A	2

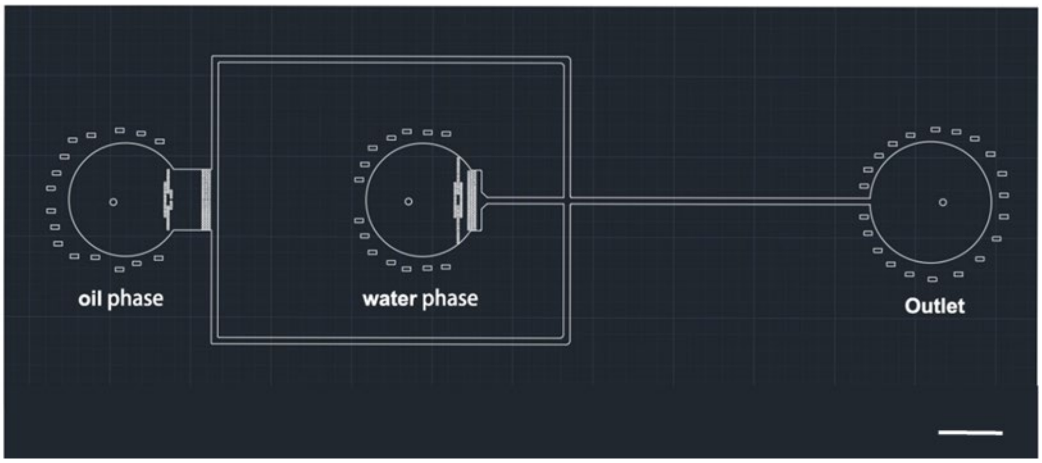

Figure S12. Design of single emulsion-making microfluidic device. A CAD file displaying the design of the microfluidic device utilised in single emulsion generation, which includes two inlets for injecting oil (continuous) and water (disperse) phases, a flow-focusing structure for creating microdroplets, and an outlet for collecting microdroplets. Channel width = 100 µm height = 50 µm. Scale bar = 100 mm.

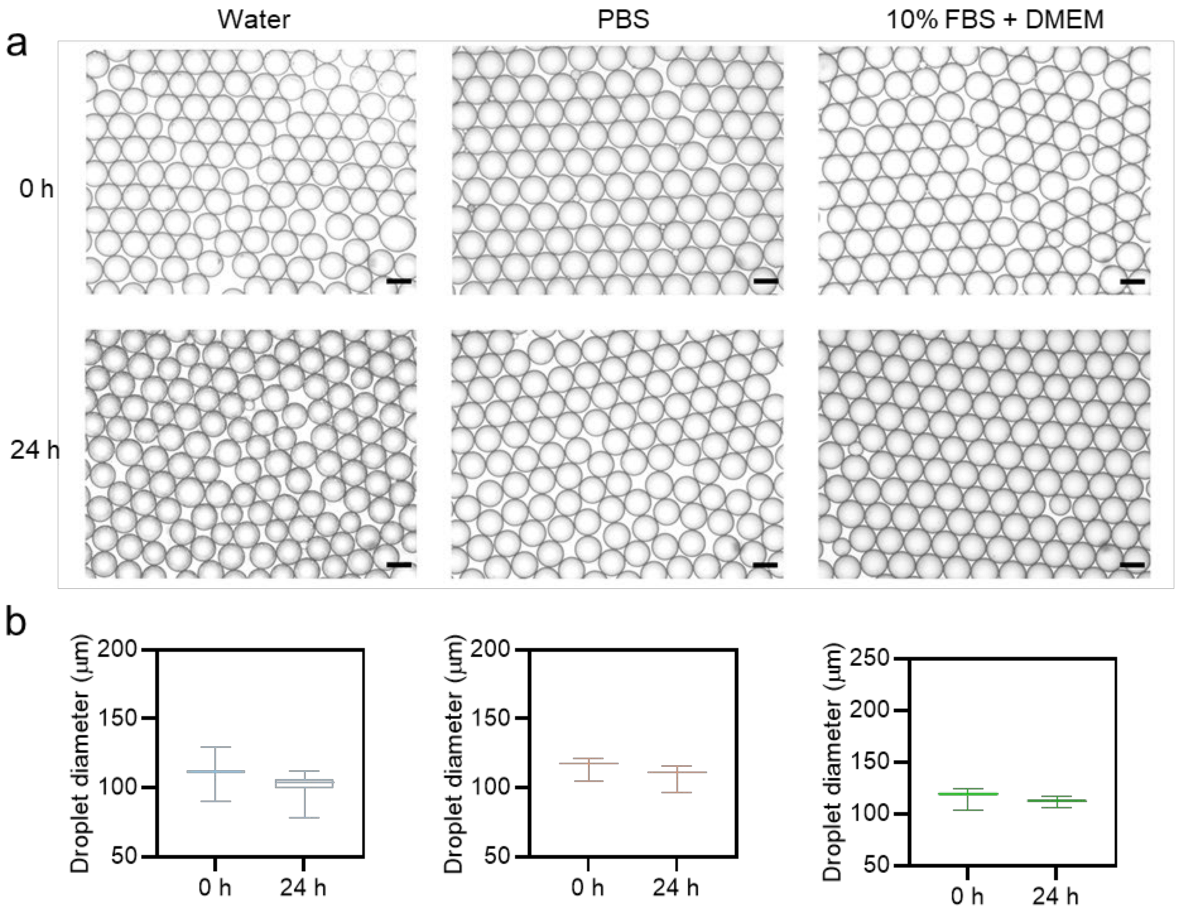

Figure S13. Droplet stability test of Pico-Surf™ surfactant. a) Micrographs displaying the size distribution of the Pico-Surf™ surfactant stabilized droplets, showing steady conditions during 24 h incubation at RT under water, PBS and DMEM + 10% FBS dispersed phase. b) Box and scatterplot of droplet size distribution after 24 h incubation at RT. The ImageJ line profiling tool was applied to measure 100 droplets to determine the mean average droplet diameter value. Scale bar, 100 μm.

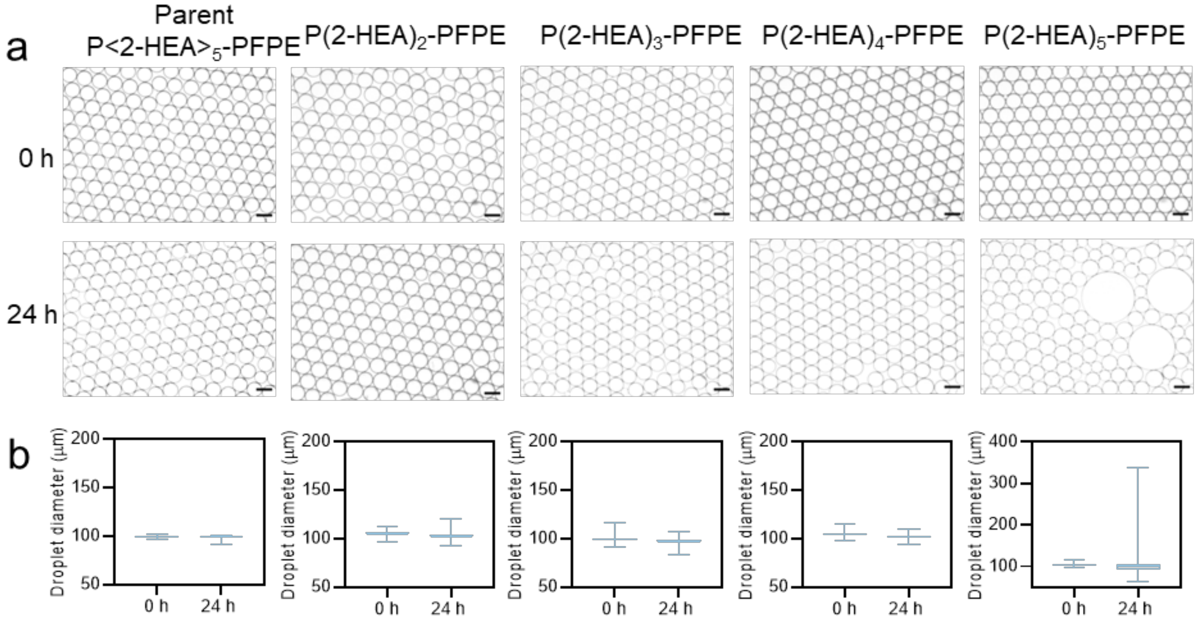

Figure S14. Droplet stability test of surfactant. a) Micrographs displaying the size distribution of the parent and fractionated P(2-HEA)$_n$-PFPE based surfactants stabilized droplets under water dispersed phase. b) Box and scatterplot of droplet size distribution after 24 h incubation at RT. The ImageJ line profiling tool was applied to measure 100 droplets to determine the mean average droplet diameter value. Scale bar, 100 µm.

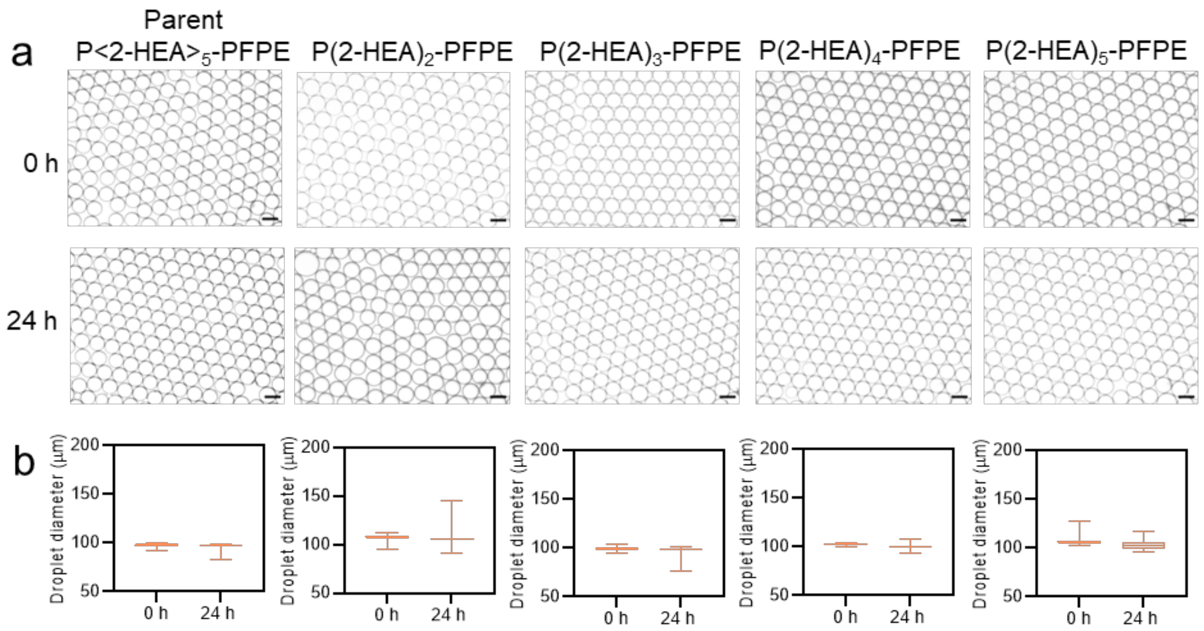

Figure S15. Droplet stability test of surfactant. a) Micrographs displaying the size distribution of the parent and fractionated P(2-HEA)$_n$-PFPE based surfactants stabilized droplets under PBS dispersed phase. b) Box and scatterplot of droplet size distribution after 24 h incubation at RT. The ImageJ line profiling tool was applied to measure 100 droplets to determine the mean average droplet diameter value. Scale bar, 100 µm.

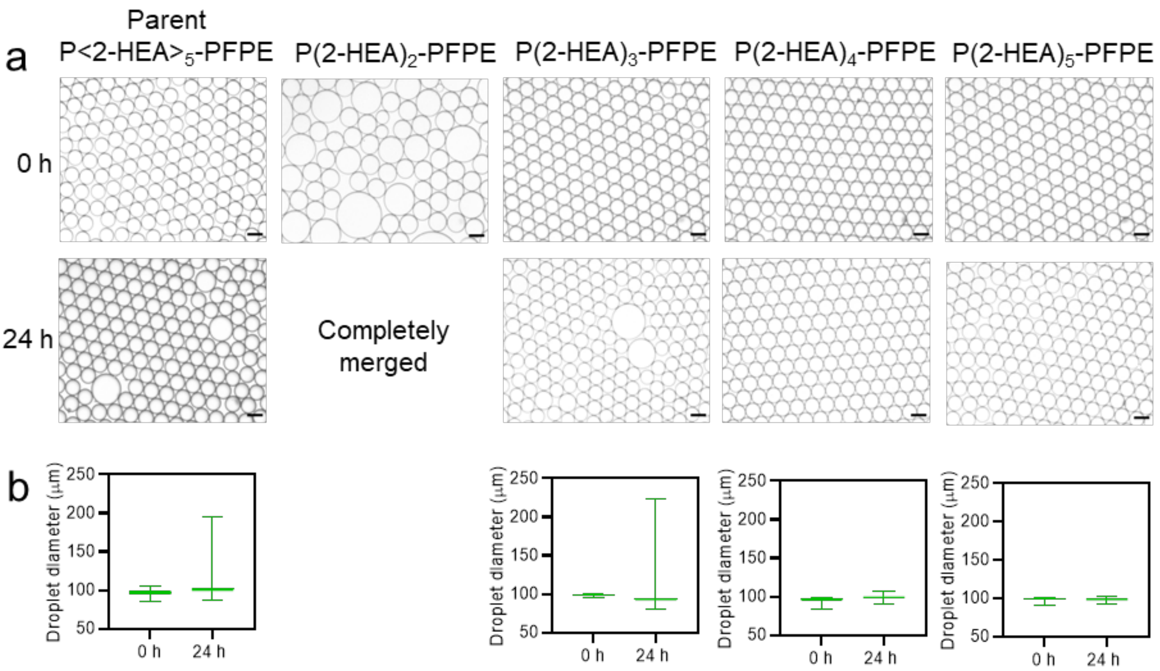

Figure S16. Droplet stability test of surfactant. a) Micrographs displaying the size distribution of the parent and fractionated P(2-HEA)$_n$-PFPE based surfactants stabilized droplets under DMEM+10% FBS dispersed phase. b) Box and scatterplot of droplet size distribution after 24 h incubation at RT. The ImageJ line profiling tool was applied to measure 100 droplets to determine the mean average droplet diameter value. Scale bar, 100 µm.

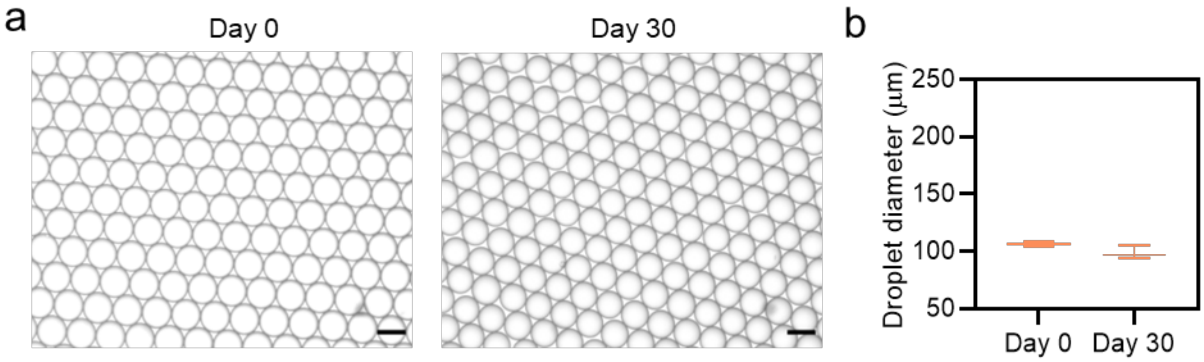

Figure S17. Droplet stability test of surfactant. a) Micrographs displaying the size distribution of the P(2-HEA)$_4$-PFPE based surfactants stabilized droplets after 30 days incubation at RT under PBS condition. b) Box and scatterplot of droplet size distribution after 30 days incubation at RT. The ImageJ line profiling tool was applied to measure 100 droplets to determine the mean average droplet diameter value. Scale bar, 100 µm

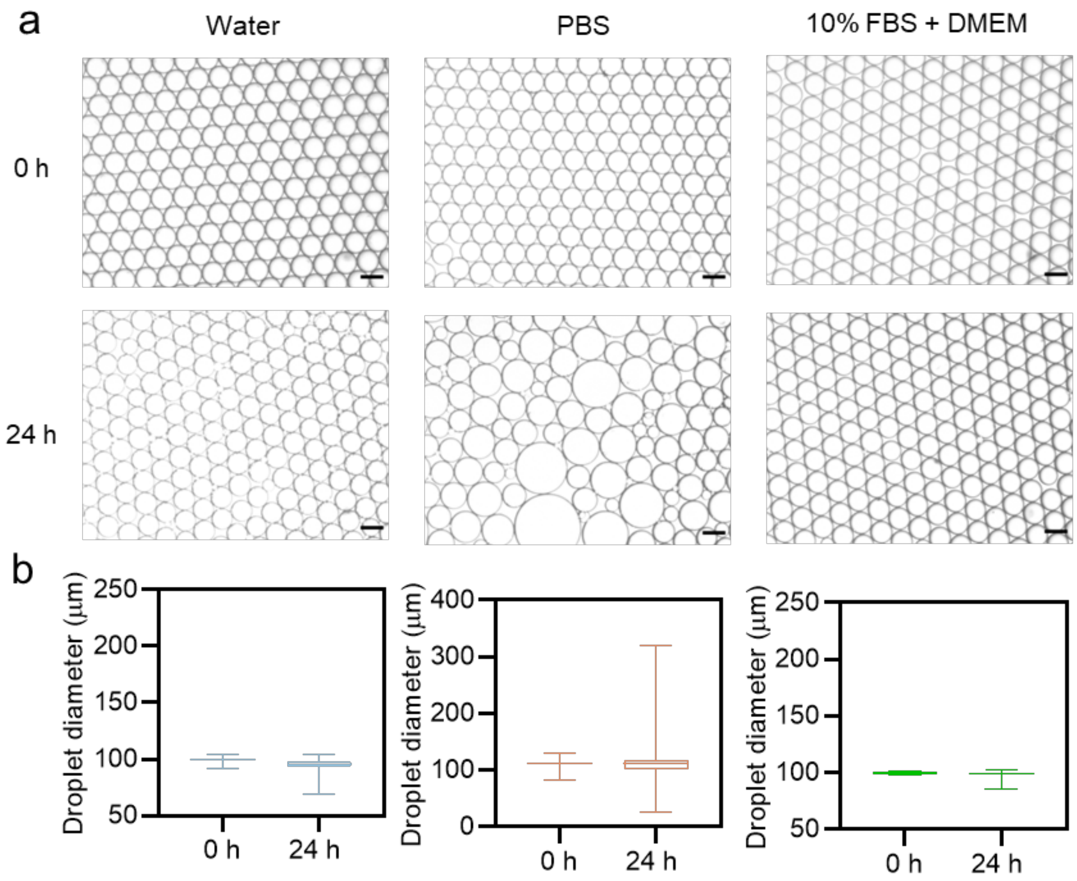

Figure S18. Droplet stability test of surfactant. a) Micrographs displaying the size distribution of the P(OEGA)$_1$-PFPE based surfactants stabilized droplets during 24 h incubation at RT under water, PBS and DMEM + 10% FBS. b) Box and scatterplot of droplet size distribution after 24 h incubation at RT. The Image J line profiling tool was applied to measure 100 droplets to determine the mean average droplet diameter value. Scale bar, 100 µm

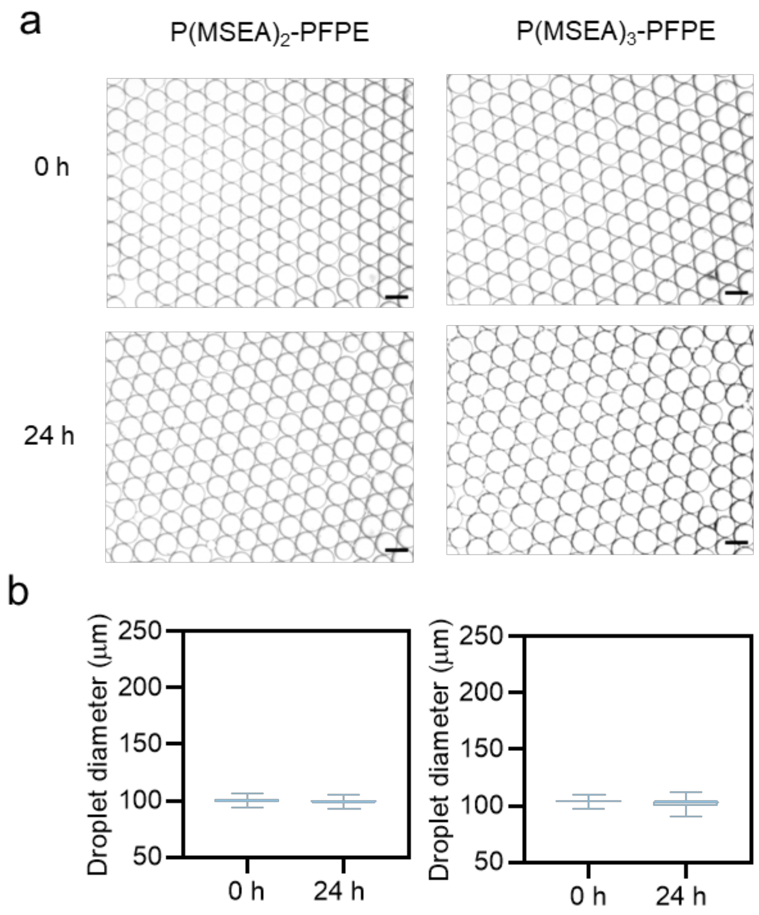

Figure S19. Droplet stability test of surfactant. a) Micrographs displaying the size distribution of fractionated $P(MSEA)_n$-PFPE based surfactants stabilized droplets under water dispersed phase. b) Box and scatterplot of droplet size distribution after 24 h incubation at RT. The ImageJ line profiling tool was applied to measure 100 droplets to determine the mean average droplet diameter value. Scale bar, 100 μm.

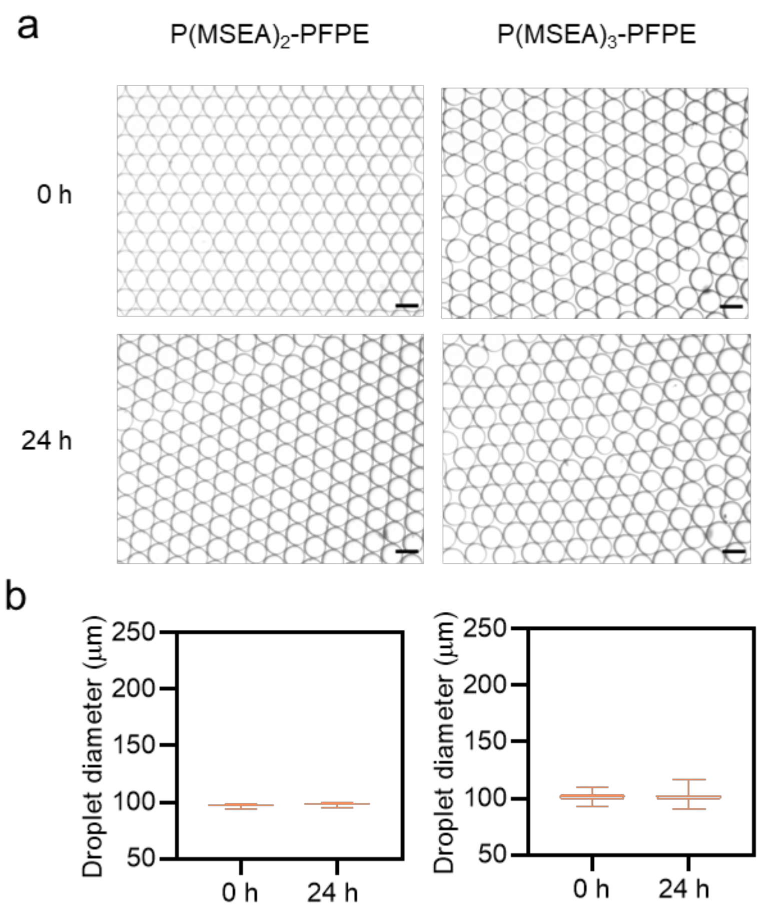

Figure S20. Droplet stability test of surfactant. a) Micrographs displaying the size distribution of fractionated P(MSEA)$_n$-PFPE based surfactants stabilized droplets under PBS dispersed phase. b) Box and scatterplot of droplet size distribution after 24 h incubation at RT. The ImageJ line profiling tool was applied to measure 100 droplets to determine the mean average droplet diameter value. Scale bar, 100 µm.

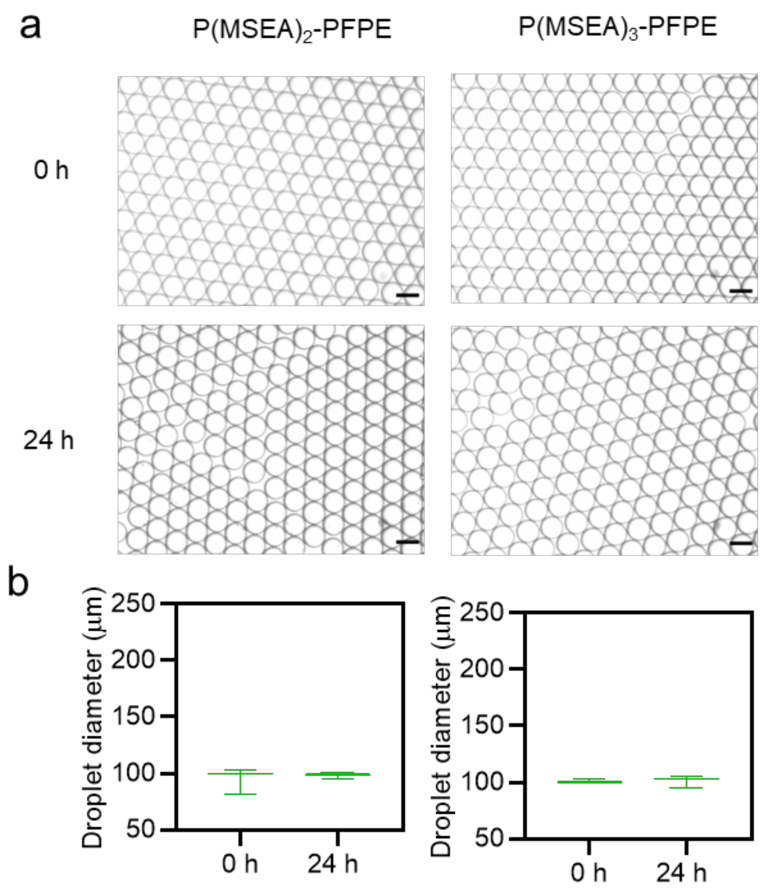

Figure S21. Droplet stability test of surfactant. a) Micrographs displaying the size distribution of fractionated P(MSEA)$_n$-PFPE based surfactants stabilized droplets under DMEM + 10% FBS dispersed phase. b) Box and scatterplot of droplet size distribution after 24 h incubation at RT. The ImageJ line profiling tool was applied to measure 100 droplets to determine the mean average droplet diameter value. Scale bar, 100 μm.

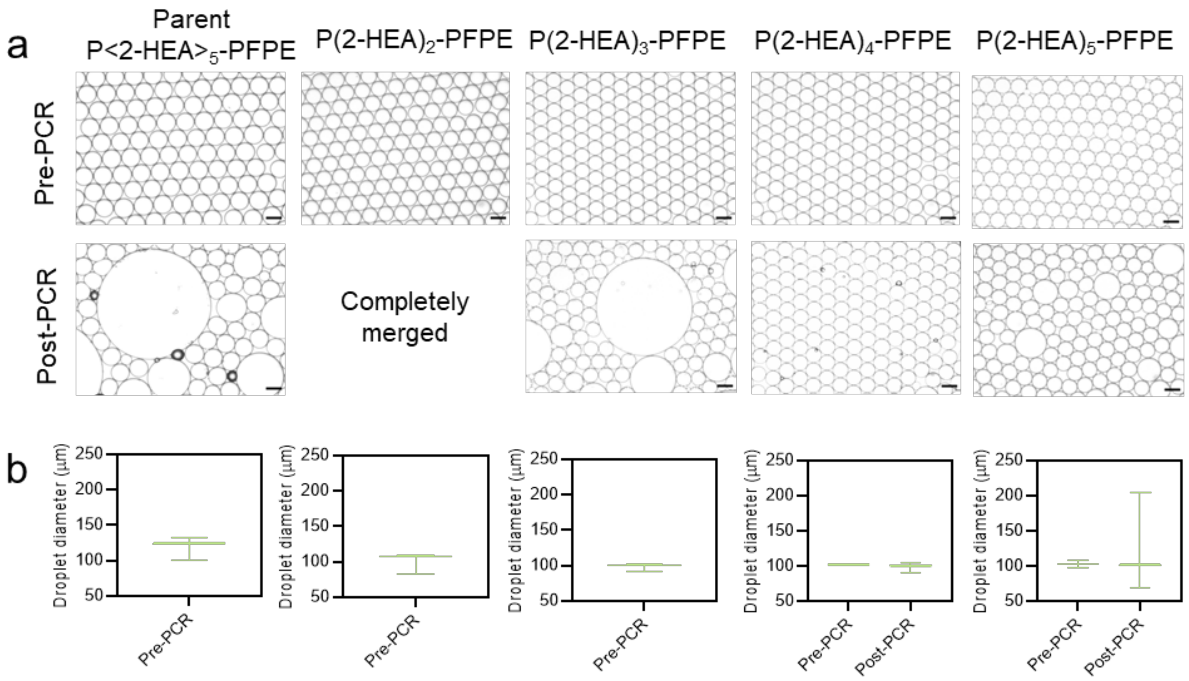

Figure S22. Thermostability of the droplets pre-PCR and post-PCR**.** a) Micrographs displaying the size distribution of the parent and fractionated P(2-HEA)$_n$-PFPE based surfactants stabilized droplets. b) Box and scatterplot of droplet size distribution before and after PCR. The ImageJ line profiling tool was applied to measure 100 droplets to determine the mean average droplet diameter value. Scale bar, 100 μm.

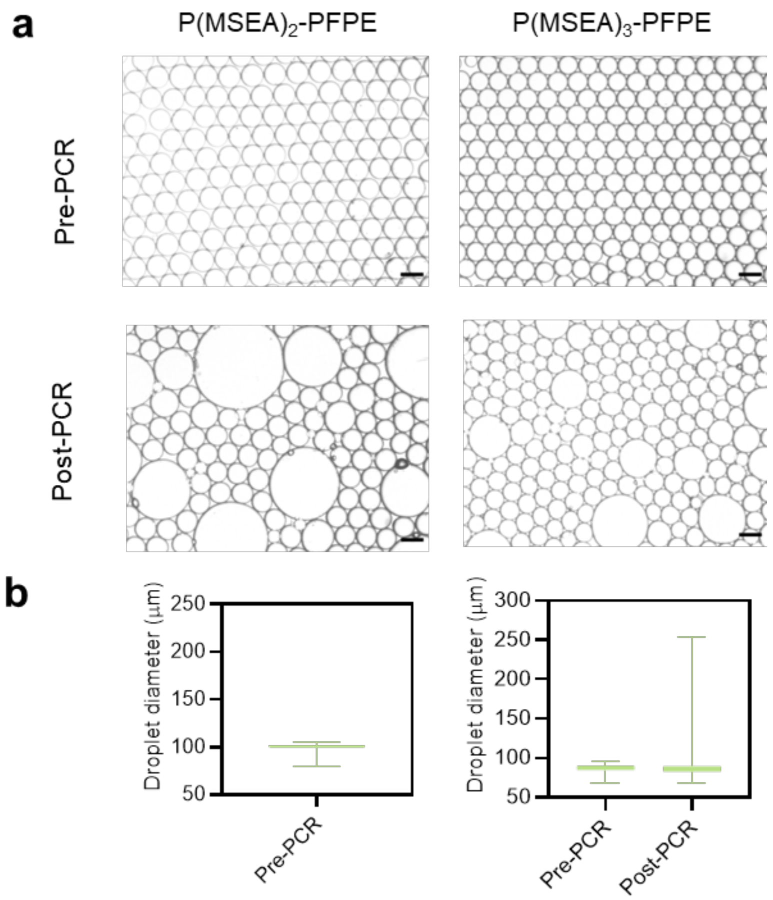

Figure S23. Thermostability of the droplets pre-PCR and post-PCR**.** a) Micrographs displaying the size distribution of the fractionated P(MSEA)$_n$-PFPE based surfactants stabilized droplets. b) Box and scatterplot of droplet size distribution before and after PCR. The ImageJ line profiling tool was applied to measure 100 droplets to determine the mean average droplet diameter value. Scale bar, 100 µm.

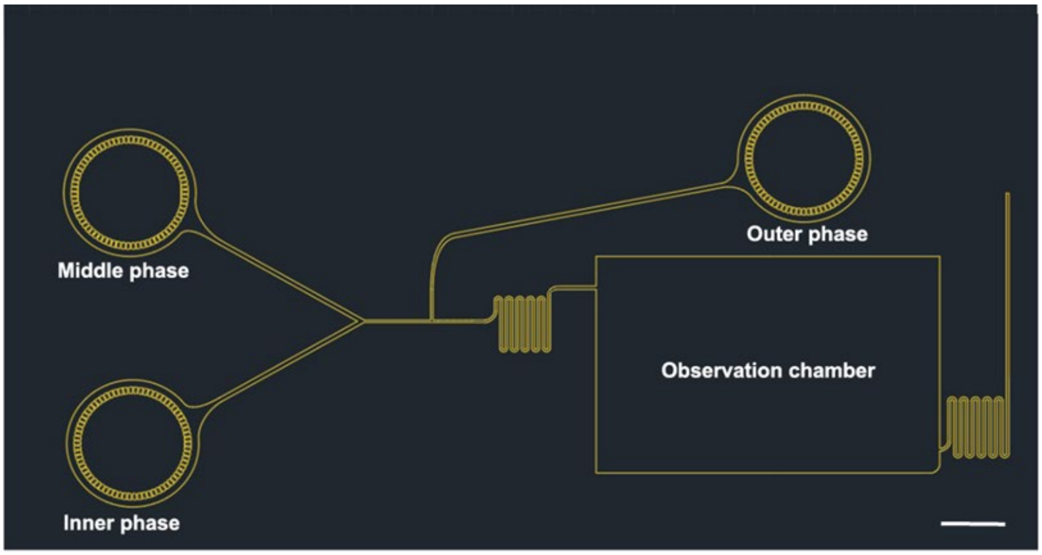

Figure S24. Design of double emulsion-making microfluidic device**.** A CAD file displaying the design of the microfluidic device utilised in double emulsion generation, which includes three inlets for injecting oil (continuous) and water (disperse) phases, a T junction for creating microdroplets, a rectangular observation chamber, and an outlet for collecting microdroplets. Channel width = 50 µm height = 50 µm.

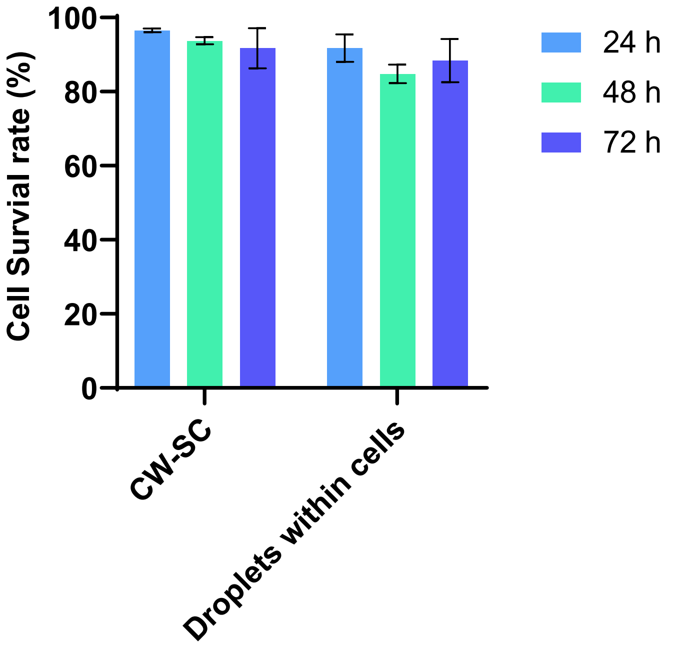

Figure S25. Cell viability was assessed under different culture conditions. As a positive control, A5 cells were cultured at a standard concentration in traditional culture wells (referred to as CW–SC) (1×10^6 cells/ml). To create cell-encapsulated droplets, we employed the P(2-HEA)$_4$-PFPE surfactant, followed by incubation at 37 °C for specified time intervals. After incubation, cells were extracted from the droplets using 1H,1H,2H,2H-perfluoro-1-octanol (PFO). Subsequently, a live/dead assay was conducted using Trypan Blue solution. Cell survival rates were estimated by counting approximately 500 to 1500 cells at designated time points. Data are presented as mean ± s.d., $n = 3$

REFERENCES

[1] C. Fu, C. Zhang, H. Peng, F. Han, C. Baker, Y. Wu, H. Ta, A. K. Whittaker, *Macromolecules* **2018**, *51*, 5875-5882.

[2] C. Zhang, S. S. Moonshi, Y. Han, S. Puttick, H. Peng, B. J. A. Magoling, J. C. Reid, S. Bernardi, D. J. Searles, P. Král, *Macromolecules* **2017**, *50*, 5953-5963.

[3] X. Xu, T. C. Williams, C. Divne, I. S. Pretorius, I. T. Paulsen, *Biotechnology for biofuels* **2019**, *12*, 1-14.
